# An antibody targeting the N-terminal domain of SARS-CoV-2 disrupts the spike trimer

**DOI:** 10.1172/JCI159062

**Published:** 2022-06-01

**Authors:** Naveenchandra Suryadevara, Andrea R. Shiakolas, Laura A. VanBlargan, Elad Binshtein, Rita E. Chen, James Brett Case, Kevin J. Kramer, Erica C. Armstrong, Luke Myers, Andrew Trivette, Christopher Gainza, Rachel S. Nargi, Christopher N. Selverian, Edgar Davidson, Benjamin J. Doranz, Summer M. Diaz, Laura S. Handal, Robert H. Carnahan, Michael S. Diamond, Ivelin S. Georgiev, James E. Crowe

**Affiliations:** 1 Vanderbilt Vaccine Center and; 2 Department of Pathology, Microbiology, and Immunology, Vanderbilt University Medical Center, Nashville, Tennessee, USA.; 3 Department of Medicine and; 4 Department of Pathology and Immunology, Washington University School of Medicine, St. Louis, Missouri, USA.; 5 Integral Molecular, Philadelphia, Pennsylvania, USA.; 6 Department of Pediatrics, Vanderbilt University Medical Center, Nashville, Tennessee, USA.; 7 Department of Molecular Microbiology, Washington University School of Medicine, St. Louis, Missouri, USA.

**Keywords:** Immunology, Virology, Adaptive immunity, Antigen, Immunoglobulins

## Abstract

The protective human antibody response to severe acute respiratory syndrome coronavirus 2 (SARS-CoV-2) focuses on the spike (S) protein, which decorates the virion surface and mediates cell binding and entry. Most SARS-CoV-2 protective antibodies target the receptor-binding domain or a single dominant epitope (“supersite”) on the N-terminal domain (NTD). Using the single B cell technology called linking B cell receptor to antigen specificity through sequencing (LIBRA-Seq), we isolated a large panel of NTD-reactive and SARS-CoV-2–neutralizing antibodies from an individual who had recovered from COVID-19. We found that neutralizing antibodies against the NTD supersite were commonly encoded by the IGHV1-24 gene, forming a genetic cluster representing a public B cell clonotype. However, we also discovered a rare human antibody, COV2-3434, that recognizes a site of vulnerability on the SARS-CoV-2 S protein in the trimer interface (TI) and possesses a distinct class of functional activity. COV2-3434 disrupted the integrity of S protein trimers, inhibited the cell-to-cell spread of the virus in culture, and conferred protection in human angiotensin-converting enzyme 2–transgenic (ACE2-transgenic) mice against the SARS-CoV-2 challenge. This study provides insight into antibody targeting of the S protein TI region, suggesting this region may be a site of virus vulnerability.

## Graphical abstract

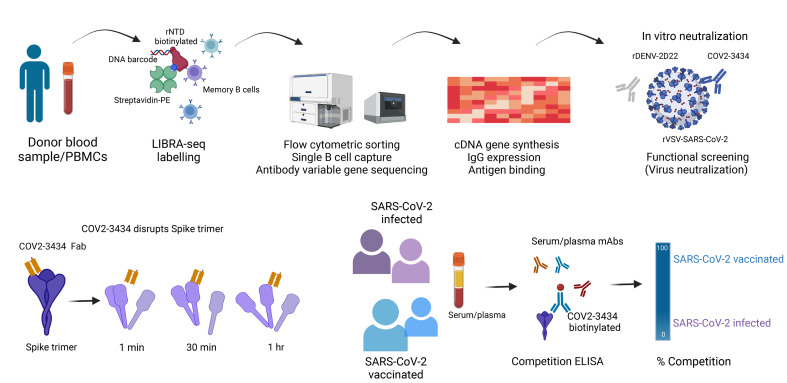

## Introduction

During the COVID-19 pandemic, more than 150 vaccine candidates were developed, but only a few have been licensed. Most licensed vaccines encode the full-length spike (S) protein, including 2 stabilizing proline mutations (S2P) of SARS-CoV-2 (1–3), and have proven effective in protecting against SARS-CoV-2 disease. Although SARS-CoV-2 vaccines have been developed at unprecedented speed, several questions remain about the efficacy and durability of the protective immunity associated with serum-neutralizing antibodies generated against the S protein. Efficacy studies are complicated by the emergence of SARS-CoV-2 variants of concern (VOC) that can escape some neutralizing antibodies. Antibodies that neutralize SARS-COV-2 VOC have been studied broadly by many groups, both in terms of their potency and structure (4–17). Similarly, it has been reported that unrelated individuals can produce genetically and functionally similar clones of antibodies (“public clonotypes”) following infection or vaccination (18–21).

The S protein’s receptor-binding domain (RBD) interacts with angiotensin-converting enzyme 2 (ACE2). In addition, the N-terminal domain (NTD) of S has been proposed to cooperate with receptors or coreceptors, such as dendritic cell–specific intercellular adhesion molecule-3–grabbing nonintegrin (DC-SIGN, also known as CD209), neuropilin-1 (NRP-1), and liver-/lymph node–specific intracellular adhesion molecule-3–grabbing nonintegrin (L-SIGN, also known as CD209L) to mediate viral attachment and enable SARS-CoV-2 infection via the established ACE2 receptor pathway (22–25). Furthermore, the NTD of the SARS-CoV-2 S protein reportedly binds biliverdin by recruitment of tetrapyrrole rings to evade neutralization of SARS-CoV-2 by some antibodies (26). SARS-CoV-2 S appears to exhibit conformational flexibility of divergent loop regions in the NTD to accommodate diverse glycan-rich host sialosides that may allow it to infect host cells with broad tissue tropism (27). Taken together, our understanding of the functional qualities of the human antibody response against NTD is incomplete. We and other groups previously identified potently neutralizing NTD-specific mAbs targeting 1 major antigenic site (7, 9–11, 28, 29). Here, using the single B cell barcoding antibody discovery technology called linking B cell receptor to antigen specificity through sequencing (LIBRA-Seq), we performed a targeted discovery of NTD-reactive antibodies from an individual who had recovered from a previous SARS-CoV-2 infection. Our results indicated that a dominant human B cell response to that significant NTD antigenic site comprises clones encoded by common variable gene segments (i.e., constitute a “public clonotype”). The scale of antibody discovery possible with LIBRA-Seq also allowed us to identify a rare clone with unusual specificity and function.

## Results

### SARS-CoV-2 infection induces a strong response against NTD and durable neutralization titers.

**Figure 1:**
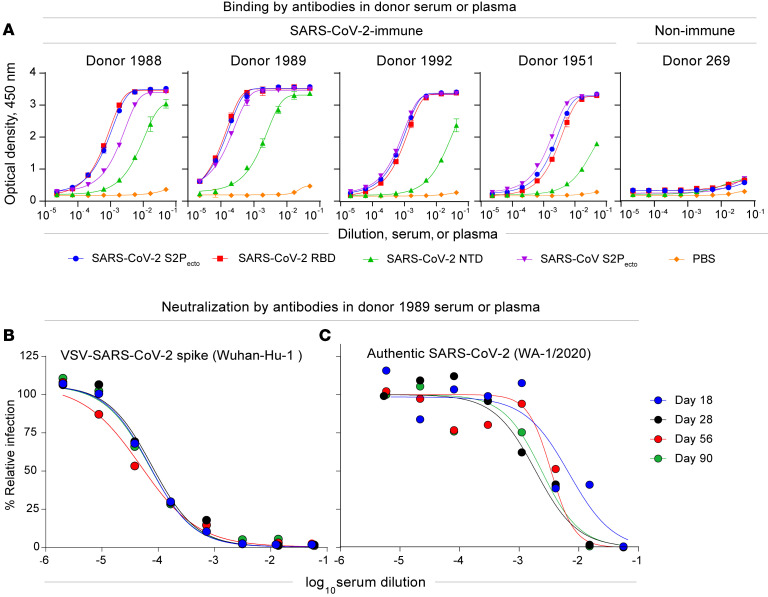
Characterization of SARS-CoV-2 antibodies in convalescent patient samples. (**A**) Serum or plasma antibody reactivity for the 4 SARS-CoV-2 convalescent patients and 1 nonimmune healthy control participant was assessed by ELISA using SARS-CoV-2 S2P_ecto_, S_RBD_, S_NTD_, SARS-CoV S2P_ecto_, or PBS. Optical density was measured with a 450 nm filter (OD_450_) using a microplate reader. Error bars indicate the SD; data are representative of at least 2 independent experiments performed in technical duplicate. (**B**) Plasma or serum neutralizing activity against the VSV-S for SARS-CoV-2 convalescent donor 1989 on days 18, 28, 56, and 90 in a RTCA neutralization assay. Data represent 2 experiments performed in technical duplicate. (**C**) Plasma or serum neutralizing activity against the WA1/2020 strain of SARS-CoV-2 for convalescent donor 1989 on days 18, 28, 56, and 90 using a FRNT. Data represent experiments performed in technical duplicate.

Peripheral blood samples were obtained following written informed consent from 4 individuals (D1988, D1989, D1995, and D1951) infected in the United States, who tested positive by PCR for SARS-CoV-2 infection, and 1 healthy donor (D269), who served as a negative control (Supplemental Table 1; supplemental material available online with this article; https://doi.org/10.1172/JCI159062DS1). We isolated plasma or serum specimens from the 5 individuals and performed serum and plasma antibody ELISA binding assays using soluble proline-stabilized S ectodomain (S2P_ecto_), RBD, or NTD protein from SARS-CoV-2 or S2P_ecto_ protein from SARS-CoV. All participants (except the negative control) had circulating antibodies that recognized each of the proteins tested, with the greatest reactivity detected against the SARS-CoV-2 S2P_ecto_, RBD, and NTD proteins (Figure 1A). The serum antibody reactivity of 1 individual (D1989) was highest against the SARS-CoV-2 NTD protein (Figure 1A). Consequently, we focused our efforts on identifying B cells from the blood samples of this individual, using sequential collections on days 18, 28, 58, and 90 after the onset of symptoms. This individual also possessed high serum neutralizing antibody titers, as determined by an assay using a chimeric vesicular stomatitis virus (VSV) displaying the SARS-CoV-2 S protein (VSV-S) in a real-time cell analysis (RTCA) method (ref. 10 and Figure 1B). The plasma neutralizing titer was good even 3 months after recovery from SARS-CoV-2 infection. To corroborate the VSV-S–based neutralization results, we also performed a serum antibody focus reduction neutralization test (FRNT) using an authentic SARS-CoV-2 strain (WA/1/2020). The authentic virus assay gave better neutralization (neutralizing antibody titer at 50% inhibition [NT_50_] = 1:258; Figure 1C) than did the VSV-based assay.

### LIBRA-Seq identifies antigen-specific B cells with high NTD specificity.

**Figure 2:**
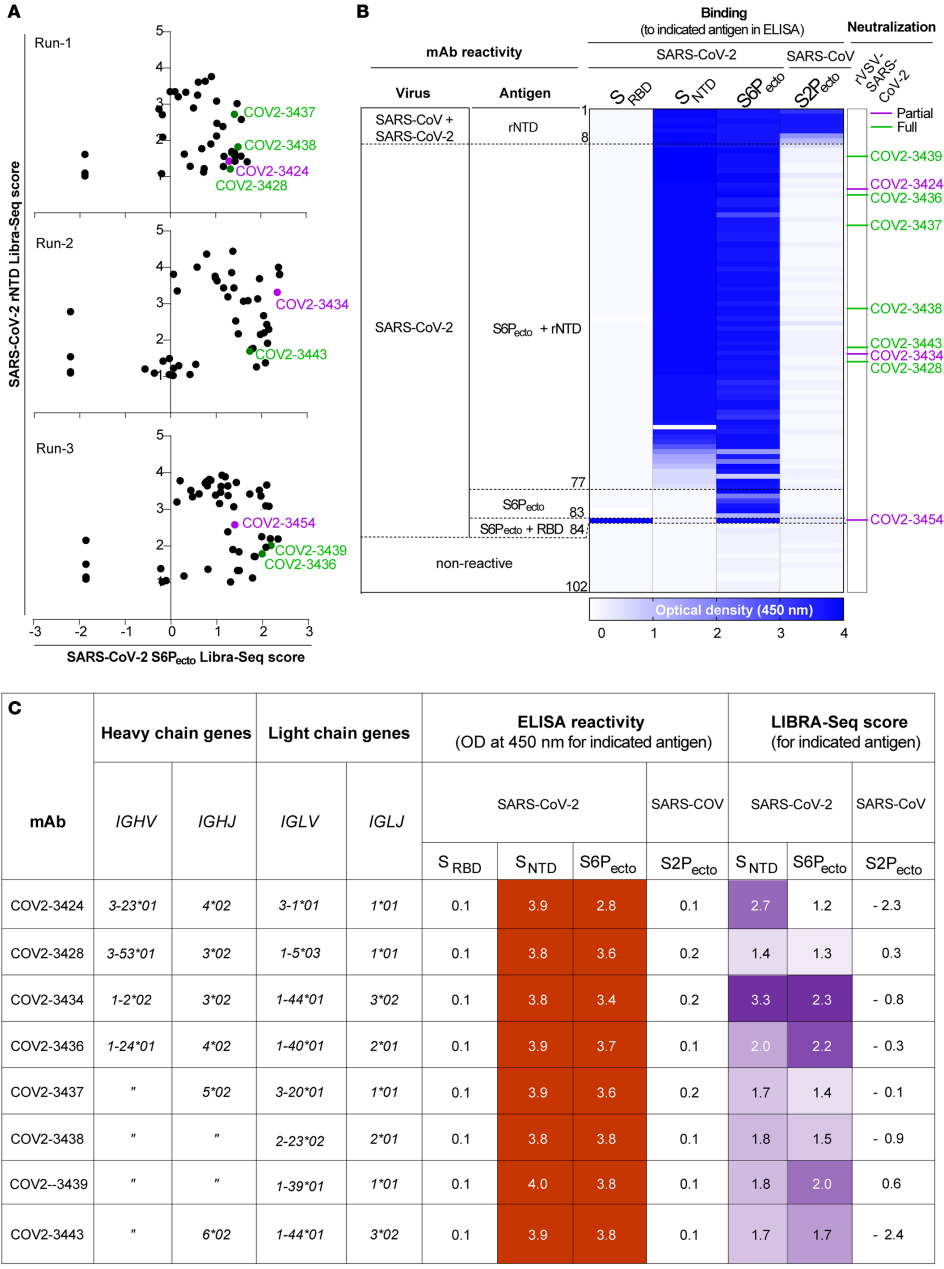
Reactivity, functional, and genetic features of 102 human mAbs identified using LIBRA-Seq. (**A**) LIBRA-Seq scores for all cells for each experiment are shown as black circles for 3 different LIBRA-Seq runs. Antibodies that demonstrated either full or partial neutralization in the high-throughput RTCA assay are highlighted in green or purple, respectively. (**B**) mAb specificity or reactivity for each of the 4 S proteins or subdomains. The figure shows a heatmap for binding of 102 mAbs expressed recombinantly, representing OD values collected at 450 nm for each antigen (range, 0.5–4.0). White indicates a lack of detectable binding, blue indicates binding, and darker blue indicates higher OD values. To the right are the antibody numbers that demonstrated either full or partial neutralization in the high-throughput RTCA assay, highlighted in green or purple, respectively. (**C**) Genetic characteristics of mAbs that demonstrated either full or partial neutralization along with their ELISA reactivity; numbers in the boxes represent OD values collected at 450 nm (range, 0.5–4.0) and LIBRA-Seq scores for each antigen. White boxes indicate no or low reactivity, and red (ELISA) and purple (LIBRA-Seq) boxes represent reactivity for the respective antigens.

Next, we used the LIBRA-Seq method (30) to identify NTD-reactive B cells. This high-throughput technology enables the determination of the B cell receptor sequence and antigen reactivity at the single-cell level. The LIBRA-Seq antigen screening library included SARS-CoV-2 S protein stabilized in a prefusion conformation (S6P_ecto_) and NTD from SARS-CoV-2 (2019-nCoV), along with antigens from other coronavirus strains and negative control antigens. We identified 347 NTD-specific B cells from individual D1989 (on day 112). We recovered 108 B cells that expressed unique V_H_-JH-CDRH3-V_L_-J_L_-CDRL3 clonotypes and gave LIBRA-Seq scores above a threshold of 1 for recombinant NTD (rNTD) (Figure 2A), and we were able to express 102 of these sequences as human mAbs. To confirm the antigen specificity predicted by the LIBRA-Seq score, we tested all expressed mAbs for binding in ELISA to recombinant monomeric RBD or NTD of SARS-CoV-2 or trimeric S6P_ecto_ of SARS-CoV-2 or trimeric S2P_ecto_ of SARS-CoV proteins. We confirmed the predicted antigen specificity for greater than 90% of the clones (Figure 2B). Most antibodies recognized the NTD protein, except for COV2-3454, which recognized the RBD protein (Figure 2B).

Additionally, using the RTCA method, we performed high-throughput neutralization assays with VSV-S and identified 9 mAbs that showed either complete (100%) or partial (50%–80%) neutralizing capacity (Figure 2B). Next, we analyzed the sequences of the variable region genes for the 102 expressed antibodies to assess the genetic diversity of the antigen-specific B cell clonotypes discovered. The expressed antibodies had diverse sequence features, including varied V- and J-gene usage, CDR3 lengths, and somatic hypermutation levels for both the heavy and light chains (Supplemental Figure 1A). After clustering these clones on the basis of the inferred immunoglobulin heavy variable (IGHV) gene, we noted that the *IGHV1-24* and *IGHV1-69* variable gene segments were used frequently in this individual’s response (Supplemental Figure 1B). Five of the 9 neutralizing mAbs are encoded by the *IGHV1-24* gene segment and are clonally unrelated (Figure 2C).

### Potently neutralizing antibodies against NTD belong to public clonotypes.

**Figure 3:**
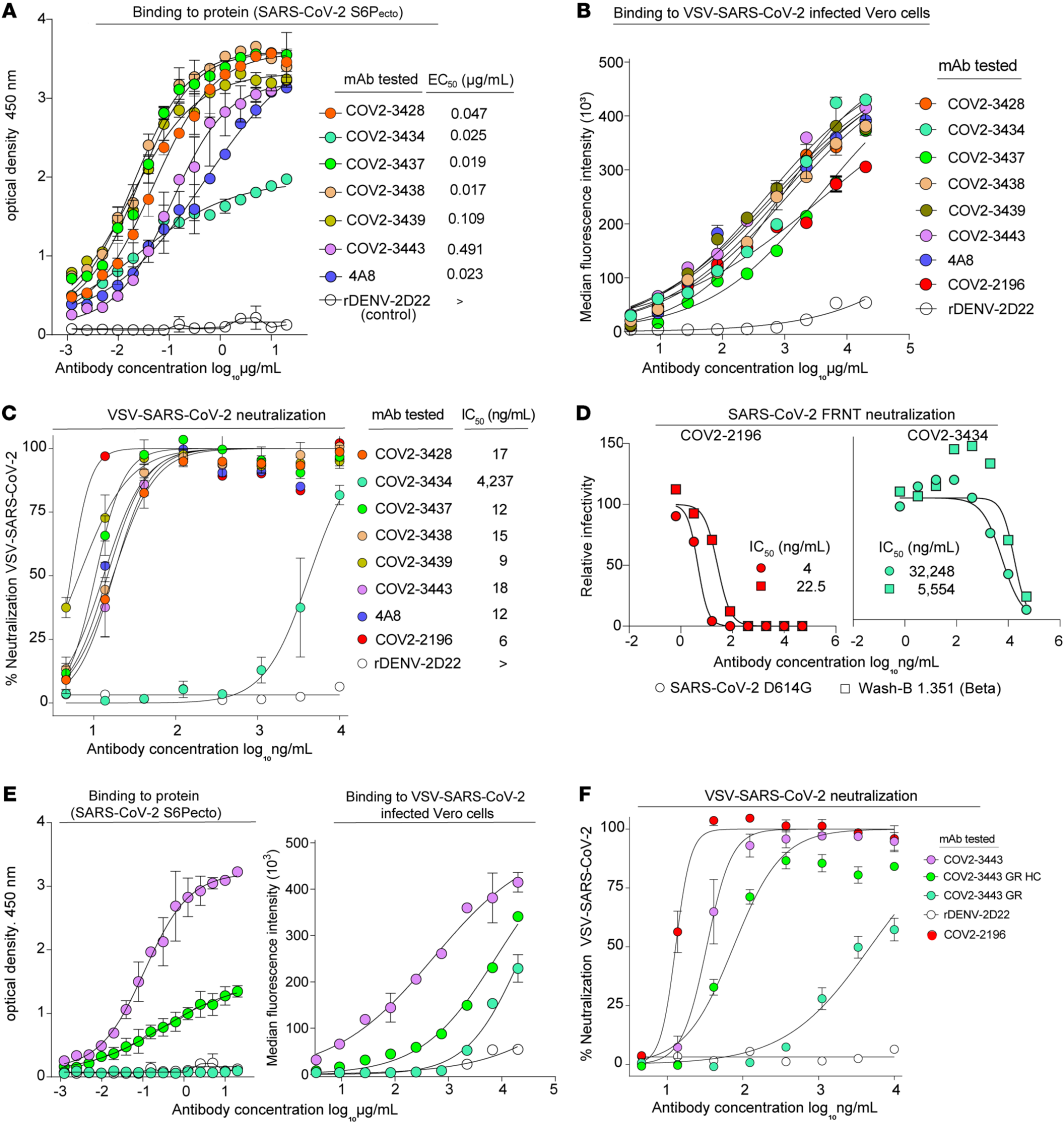
Activity of neutralizing mAbs against SARS-CoV-2. (**A**) ELISA binding to SARS-CoV-2 S6P_ecto_ protein was measured by absorbance at 450 nm. Antibody concentrations starting at 20 μg/mL were used and titrated 2-fold. Calculated EC_50_ values are shown on the graph. Error bars indicate the SD; data represent at least 2 independent experiments performed in technical duplicate. (**B**) Binding to the surface of VSV-S–infected Vero cells was measured by flow cytometry, and MFI values were determined for dose response binding curves. Antibodies were diluted 3-fold starting from 20 μg/mL. Data represent 2 experiments performed in technical triplicate. (**C**) VSV-S neutralization curves for mAbs that were expressed after high-throughput RTCA neutralization conformation. Calculated IC_50_ values are shown on the graph. Error bars indicate the SD; data represent at least 2 independent experiments performed in technical duplicate. (**D**) Neutralization curves for COV2-3434 or COV2-2196 against SARS-CoV-2 virus. Calculated IC_50_ values are shown on the graph. Error bars indicate the SD; data represent at least 2 independent experiments performed in technical duplicate. (**E**) To determine GR COV2-3443 antibody reactivity and functional activity, ELISA binding to SARS-CoV-2 S6P_ecto_ protein was measured by absorbance at 450 nm. Binding to the surface of VSV-S–infected Vero cells was measured by flow cytometry, and MFI values were determined for dose response binding curves. (**F**) VSV-S neutralization curves for GR COV2-3443 antibody. Error bars indicate the SD; data represent at least 2 independent experiments performed in technical duplicate.

Next, we determined the binding activity of the panel of NTD-reactive neutralizing antibodies. Using serial dilution studies, we determined the EC_50_ for binding to the S6P_ecto_ trimer protein, in comparison with a known NTD-reactive mAb (4A8) or a negative control dengue-specific antibody (rDENV-2D22). NTD-reactive neutralizing antibodies exhibited varied binding profiles with a diverse range of EC_50_ values (Figure 3A). We also tested the panel of antibodies for binding to cell-surface–displayed S protein on SARS-CoV-2–infected cells according to the gating strategy shown in Supplemental Figure 2. Unexpectedly, the NTD-targeting mAbs stained infected cells with greater intensity (higher median fluorescence intensity [MFI]) than did a previously described high-affinity, RBD-reactive potently neutralizing mAb (COV2-2196) (ref. 6 and Figure 3B). We also determined the inhibitory potency for representative mAbs in the quantitative VSV-S–based neutralization assay (Figure 3C). These results confirmed that the LIBRA-Seq technology efficiently identifies mAbs with the correct antigen specificity and that some of the NTD-reactive mAbs potently neutralize VSV-S infection based on RTCA neutralization (6, 10). Next, we chose COV2-3434 for further study with an FRNT, as it showed a distinct phenotype in both binding and rVSV neutralization. We performed FRNTs for mAb COV2-3434 using the strains SARS-CoV-2 D614G and chimeric strains expressing the B.1.351 (Beta) spike on a WA1/2020 background (Wash-B 1.351; ref. 4). COV2-3434 neutralized both strains of SARS-CoV-2 in a dose-dependent manner, with IC_50_ values of 5.5 and 32 μg/mL, respectively (Figure 3D). A comprehensive analysis of antibody variable gene sequences for SARS-CoV-2 human mAbs revealed that the *IGHV1-24* gene segment is frequently used in vaccinated or convalescent individuals when targeting the NTD (Supplemental Table 2 and Supplemental Figure 3). Nevertheless, the clones recovered here were unique, with diverse gene usage for both heavy and light chains. There was no bias for a particular HCDR3 length that confers NTD specificity. Additionally, the *IGHV1-69* and *IGHV3-53* gene segments were overrepresented in both RBD- and NTD-specific antibodies isolated from convalescent subjects. Of note, the *IGHV1-69* gene–encoded antibodies that reacted with NTD did not neutralize VSV-S, and the other V_H_ genes used (*IGHV1-2*, *IGHV3-23*, and *IGHV3-53*) encoded clones with only moderate neutralizing capacity. Thus, the most potently neutralizing, NTD-reactive antibodies isolated here were encoded by *IGHV1-24*.

To determine whether the functional activity of *IGHV1-24*–encoded antibodies identified in this study was due to germline-encoded reactivity or the result of somatic mutations, we engineered “germline-revertant” (GR) recombinant antibodies that were reverted at residues that differed from the germline gene segments either in the heavy chain (GR-HC) or in both heavy and light chains (GR). After alignment of the sequences of *IGHV1-24–*encoded clones with the germline gene segment *IGHV1-24*, we chose the mAb COV2-3443 for further study, as it was the antibody with the fewest somatic mutations. We tested whether the GR mAb shared similar functional properties with its somatically mutated counterparts for binding to the S protein or VSV-S neutralization. The COV2-3443 GR-HC mAb retained some binding and neutralization capacity, whereas COV2-3443 GR completely lost its binding and neutralization capacity, suggesting that the functional activities required some or all of the somatic mutations present in the matured antibody (Figure 3, E and F).

### COV2-3434 maps to a distinct site from the NTD supersite.

**Figure 4:**
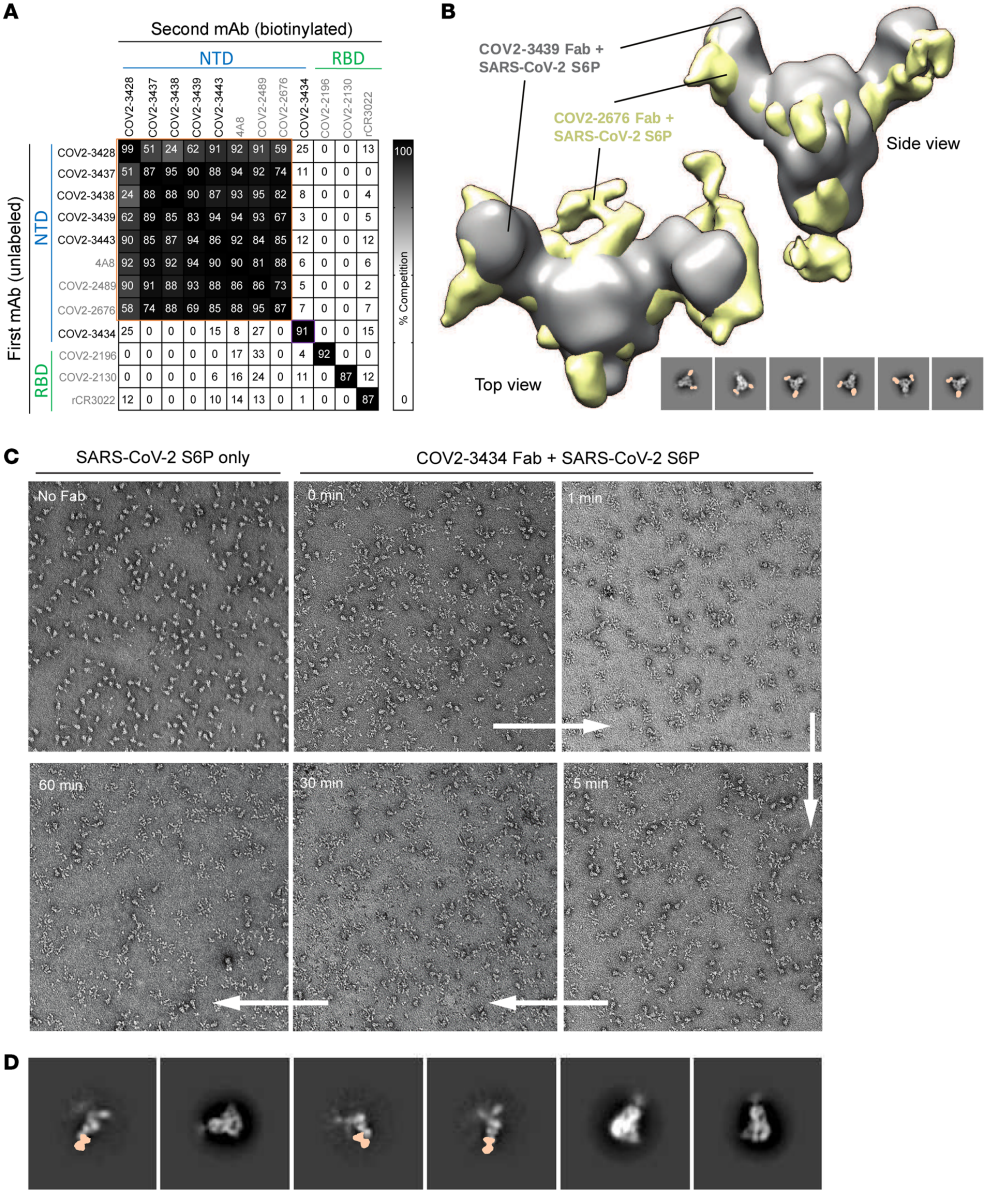
Epitope identification and structural characterization of COV2-3439 and COV2-3434 antibodies. (**A**) Competition of the panel of neutralizing mAbs with the previously mapped antibodies COV2-2130, COV2-2196, COV2-2676, COV2-2489, r4A8, and rCR3022. Unlabeled antibodies that were applied to the antigen first are indicated on the left, whereas biotinylated antibodies that were added to antigen-coated wells second are listed across the top. The number in each box represents the percentage of competition binding of the biotinylated antibody in the presence of the indicated competing antibody. Heatmap colors range from dark gray (100% binding of the biotinylated antibody) to white (0% or no binding of the biotinylated antibody). The experiment was performed in biological replicate. A biological replicate from a representative single experiment is shown. (**B**) nsEM of the SARS-CoV-2 S6P_ecto_ protein in complex with COV2-3439 Fab. Shown are the side view and top view of superimposed 3D volume COV2-3439 Fab–S6P_ecto_ closed trimer (S protein model PDB:7JJI) complexes, as visualized by nsEM for the COV2-2676 Fab model in gold and the COV2-2489 Fab model in gray. At the bottom, negative-stain 2D classes of SARS-CoV-2 S protein incubated with COV2-3439 Fab are shown. Data are from a single experiment (detailed collection statistics are provided in Supplemental Table 3). (**C**) Morgagni images of SARS-CoV-2 S6P_ecto_ protein only, immediately after COV2-3434 Fab was added to the SARS-CoV-2 S6P_ecto_ trimer, incubated for 1, 5, or 30 minutes or 1 hour, and then placed on a nsEM grid. (**D**) Negative-stain 2D classes of SARS-CoV-2 S6P_ecto_ protein only or COV2-3434 Fab with a monomer of SARS-CoV-2 S6P_ecto_ protein (based on the density surrounding the Fab).

We next defined antigenic sites on the NTD by competition-binding analysis. We used SARS-CoV-2 6P_ecto_ protein to screen for NTD-reactive neutralizing mAbs that competed for binding with each other or with the previously described NTD-reactive mAbs COV2-2676 and COV2-2489, which recognize known epitopes on the NTD protein (10). We also used the previously described RBD-reactive neutralizing (COV2-2196 and COV2-2130) or non-neutralizing (rCR3022) mAbs as controls. We identified 2 groups of competing mAbs in the NTD (Figure 4A). The first group competed for binding to the known NTD supersite, which we and others have described previously (7, 9–11). The second competition group contains a single mAb (COV2-3434) that bound to a site distinct from that of the supersite of all other NTD-reactive mAbs (Figure 4A). We also tested competition of the COV2-3434 mAb with the recently reported antibody 5 to 7, which binds a hydrophobic site on NTD. Our mAb COV2-3434 did not compete for binding with mAb 5 to 7 either on SARS-CoV2-6P_ecto_ or NTD; furthermore, COV2-3434 also did not block ACE2 from binding to the S protein, revealing that the COV2-3434 site is unique (Supplemental Figure 4).

### COV2-3434 exhibits trimer-disrupting properties.

To further probe the binding sites for these mAbs, we used negative-stain electron microscopy (nsEM) to image a stabilized trimeric form of the ectodomain of the S protein (S6P_ecto_ trimer) in a complex with Fab fragment forms of COV2-3439 or COV2-3434. We chose COV2-3439 as a representative mAb from the first competition group, as it was the most potently neutralizing antibody against VSV-S. COV2-3439 bound to the NTD and recognized the “closed” conformational state of the S6P_ecto_ trimer. We confirmed that the COV2-3439 antibody binds to the previously noted antigenic supersite on the NTD of the S6P_ecto_ trimer by overlaying the nsEM maps of the COV2-3439 Fab-S protein complex with our previously published COV2-2676 Fab-S complex (Figure 4B).

Unexpectedly, we did not observe intact S protein trimers following a 1-hour incubation with saturating concentrations of COV2-3434 Fab fragments. Shorter incubation times with Fabs (1, 5, or 30 min) showed more intact trimers in the grids (Figure 4C). Representative 2D images revealed that Fabs were bound to the S protomers, suggesting that Fabs recognize an epitope that is not present or accessible on an intact S trimer (Figure 4D). Although the 2D images are revealing, we could not create reconstructions of the Fab protomers, since there were very limited views of the complexes. The data are consistent with a trimer disruption mechanism in which binding of the COV2-3434 Fab to a partially occluded epitope drives the disruption of the S protein trimer. We also performed an S1 shedding experiment to see whether COV2-3434 works through the mechanism described for some RBD-binding antibodies (31, 32). Unlike S2H97 or COV2-2196, COV2-3434 did not shed S1 either on CHO-K1 cells stably expressing the prototypic SARS-CoV-2 spike protein or on the authentic virus (Supplemental Figure 5A).

**Figure 5:**
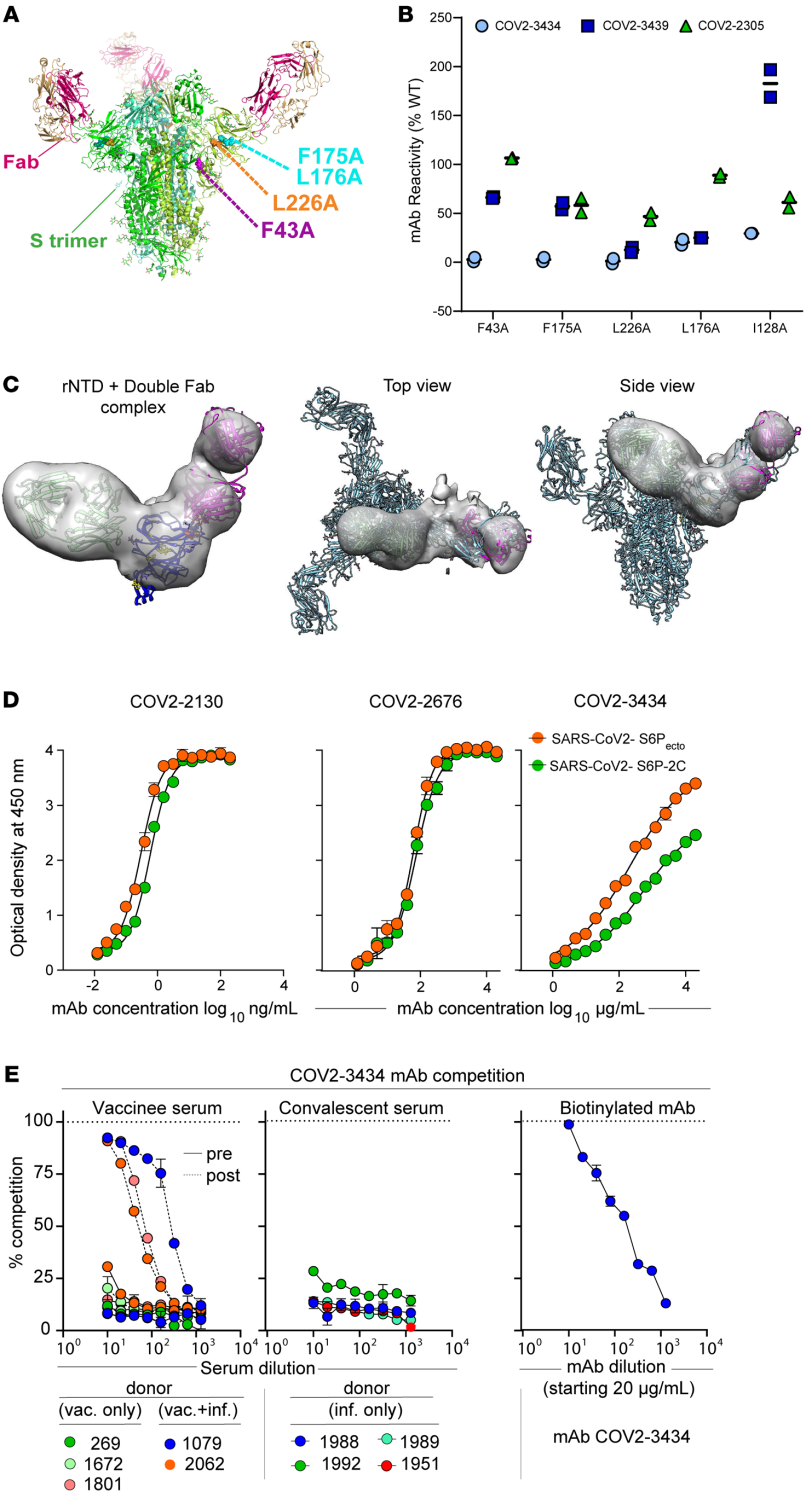
Structural characterization of the trimer-disrupting antibody COV2-3434. (**A**) Residues identified as important for COV2-3434 binding are highlighted as spheres on the S protein structure: Protein Data Bank (PDB) 7L2C (green ribbons), F43 (magenta), F175 and L176 (cyan), and L226 (orange). Residues critical for COV2-3434 binding were identified from binding screens of an alanine-scanning mutagenesis library of NTD. (**B**) mAb binding values for COV2-3434, COV2-3439, and control anti-NTD mAb COV2-2305 are shown at SARS-CoV-2 S protein clones identified as critical for MAb binding. mAb reactivities for each mutant are expressed as a percentage of binding to wild-type S protein, with ranges (half of the maximum minus minimum values). Two replicate values were obtained for each experiment. (**C**) nsEM of SARS-CoV-2 rNTD protein in complex with COV2-3439 and COV2-3434 Fabs. Shown are the top view and side view of superimposed 3D volume COV2-3434 Fab–COV2-3439 Fab–SARS-CoV-2 rNTD complexes as visualized by nsEM aligned to the S protein of SARS-CoV-2 in complex with 4A8 (PDB: 7C2L) Data are from a single experiment (detailed collection statistics are provided in Supplemental Table 3). (**D**) ELISA binding to SARS-CoV-2 S6P_ecto_ or SARS-CoV-2 S6P-2C was measured by absorbance at 450 nm. The COV2-2130 starting concentration was 200 ng/mL, the COV2-2676 and COV2-3434 starting concentrations were 20 μg/mL, and mAbs were titrated 2-fold. Calculated EC_50_ values are shown on the graph. Error bars indicate the SD; data represent at least 2 independent experiments performed in technical duplicate. (**E**) Measurement of serum antibody competition with TI antibody COV2-3434 in individuals before or after SARS-CoV-2 mRNA vaccination. Competition-binding ELISA curves for COV2-3434 with human serum from convalescent or vaccinated donors. Competition-binding experiments were performed for each sample in triplicate and repeated in at least 2 independent experiments. One representative experiment is shown. For all competition-binding curves, data points indicate the mean and error bars indicate the SD. vac., vaccinated; inf., infected.

We next defined the COV2-3434 and COV2-3439 epitopes at the amino acid level using 2 complementary methods: alanine-scanning loss-of-binding experiments and cell-surface S protein display. Screening of the NTD alanine scan library identified the primary residues F43, F175, L176, and L226 as critical for binding of COV2-3434 (Figure 5A), whereas for COV2-3439, residues R102, Y145, K147, W152, R246, Y248, P251, and G252 were identified (Supplemental Figure 5B). Although some alanine mutants affected binding of the control NTD-reactive mAb COV2-2305 up to 25%, this could be due to residues I128, F175, and L176 being fully buried (L226A is partially buried) within the hydrophobic core of the NTD tertiary fold, and alanine substitutions could very likely result in structural changes and alterations of epitopes (Figure 5B). As an alternative approach to learn more about the epitope recognized by this trimer-disrupting antibody, we generated complexes of the NTD subdomain with Fabs of COV2-3434 and COV2-3439. Interestingly, in nsEM, we noticed that the COV2-3434 Fab bound NTD at a 90° angle to that of the supersite-binding COV2-3439 Fab (Figure 5C). Moreover, when we overlaid this double Fab plus rNTD complex onto that of the trimeric spike complex (7C2L model), COV2-3434 Fab tangentially clashed with an interface of RBD and NTD (Figure 5C). Modeling of double Fab and NTD complexes onto the spike monomer, dimer, and trimer when RBD was open enabled us to locate Fab binding more precisely and suggested that the epitope recognized by COV2-3434 is occluded (Supplemental Figure 6). Recently, it was reported that the NTD of SARS-CoV-2 spike binds biliverdin and polysorbate 80 by recruitment of tetrapyrrole rings to evade antibody neutralization. However, our neutralization assays in the presence of biliverdin or polysorbate 80 did not affect COV2-3434 neutralization of VSV-S (Supplemental Figure 7), again suggesting this epitope is distinct. Additional structural studies are needed to determine the structural basis for the trimer-disrupting phenotype of mAbs binding to this epitope.

The S protein exhibits high flexibility between domains and can exist in different conformations, allowing the immune system to target distinct epitopes and structural states (33). Henderson et al. showed that conformations of the S protein can be controlled via rational design using expressed soluble ectodomains of the S proteins, in which the 3 RBDs are either locked in the all-RBDs “down” position (S6P_ecto_-2C) or adopt “up” state (S6P_ecto_) conformations (33). We hypothesized that the COV2-3434 binding site is accessible only when the RBD adopts an up state conformation of S6P_ecto_. To test this model, we quantified the binding of COV2-3434 to S6P_ecto_ or S6P_ecto_-2C proteins by ELISA. For comparison, we also included a mAb that binds to RBD in either the up or down conformational state (COV2-2130), a mAb that binds to NTD (COV2-2676), and the negative control dengue mAb DENV-r2D22. As expected, the binding of COV2-3434 to S6P_ecto_-2C protein was reduced, confirming that the epitope is cryptic and only accessible when at least 1 RBD is in its up conformation (Figure 5D).

### SARS-CoV-2 mRNA vaccines can induce trimer-disrupting antibodies.

Although we identified a new antigenic site by isolating COV2-3434 from a SARS-CoV-2 convalescent donor, it is uncertain if this class of antibodies forms a major part of the humoral immune response to the S protein trimer. To address this question, we performed a competition-binding ELISA with serum antibodies and COV2-3434. Serum antibodies from each of 4 naturally SARS-CoV-2–infected individuals or from each of 5 individuals before or after SARS-CoV-2 mRNA vaccination were tested. We observed up to 90% serum antibody competition with COV2-3434 in 3 of the donors tested following vaccination, indicating that in some individuals SARS-CoV-2 mRNA vaccination generates high levels of antibodies specific for the S protein trimer interface (TI) or antibodies that compete with TI antibodies (Figure 5E). In contrast, we did not observe this level of competition with COV2-3434 in serum from the convalescent donors. Taken together, these results suggest that S protein TI antibodies may be more common in the serum of vaccinated individuals than in infected individuals. The reason this class of antibodies was observed in the serum of vaccinees but not convalescent individuals is not clear, although engineered vaccine S antigen differs from the natural S protein in that the “prefusion” S conformation was stabilized in the vaccine construct by mutagenesis.

### COV2-3434 inhibits VOC and confers partial protection against SARS-CoV-2 infection.

**Figure 6:**
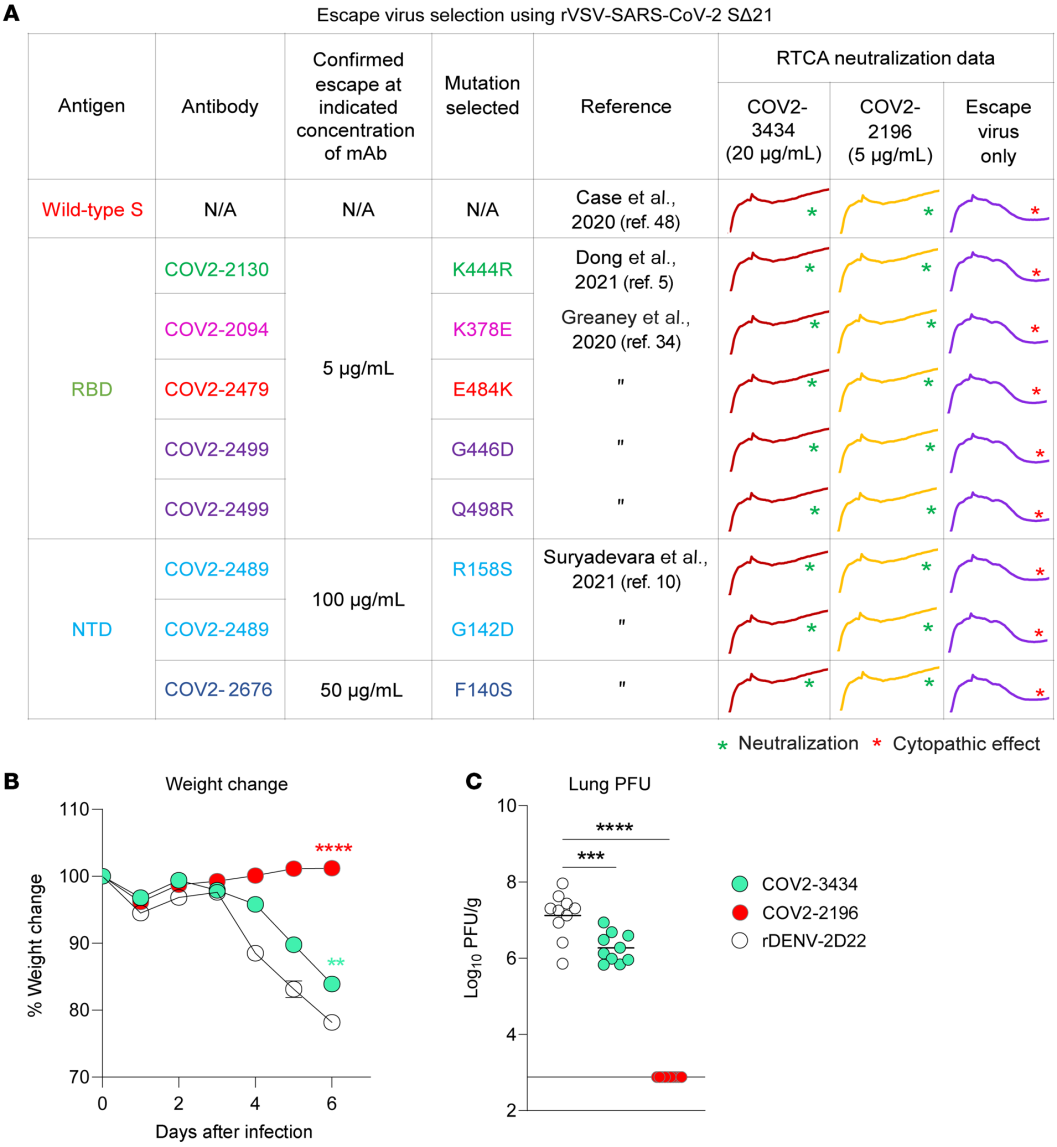
Escape virus neutralization and protection in K18 hACE2–transgenic mice by the trimer-disrupting antibody COV2-3434. (**A**) Neutralization of mAb escape viruses selected by the RBD-specific mAbs COV2-2479 (red), COV2-2130 (green), COV2-2094 (magenta), or COV2-2499 (purple) and the NTD-specific mAbs COV2-2676 (blue) or COV2-2489 (cyan) and with VSV-S by COV2-3434 or COV2-2196 (positive control). The mutations selected by those mAbs are listed with the references. In the right column, the RTCA curves show neutralization of those escape viruses; the asterisk indicates a lack of neutralization in wells with only virus and no antibody. (**B**) Eight-week-old male K18-hACE2–transgenic mice were inoculated via the i.n. route with 10^4^ FFU of SARS-CoV-2 (WA1/2020 D614G). One day prior to virus inoculation, mice were given a single 200 μg (~10 mg/kg) dose of COV2-3434 or COV2-2196 by i.p. injection. Weight change was monitored daily. Data are from 2 independent experiments (*n =* 10 per group). **P < 0.01 and ****P < 0.0001, by 2-way ANOVA. Error bars represent the SEM. (**C**) On day 6 after infection, lungs were collected and assessed for infectious viral burden by plaque assay. PFU/g is shown. Bars indicate the mean viral load; the dotted line indicates the limit of detection of the assay. Data are from 2 independent experiments (*n =* 10 per group). ***P < 0.0001 and ****P < 0.0001, by 1-way ANOVA.

Identification of neutralizing mAbs that bind to distinct antigenic sites on S proteins might help to avoid escape from neutralization by VOC. To address this idea, we used VSV-S viruses expressing SARS-CoV-2 S protein variants that were resistant to neutralization by the RBD-specific antibodies COV2-2479, COV2-2499, and COV2-2130 (34) or resistant to the NTD-specific antibodies COV2-2676 and COV2-2489 (10). The COV2-3434 mAb neutralized all escape VSV viruses at the higher concentration tested (Figure 6A).

We next assessed the ability of COV2-3434 to protect K18-hACE2–transgenic mice following viral challenge with SARS-CoV-2 (35–37). One day prior to virus inoculation, we passively transferred approximately 10 mg/kg (200 μg/mouse) COV2-2196 (RBD-specific), COV2-3434 (NTD-specific), or DENV-r2D22 (negative control) mAbs. Mice that received r2D22 lost more than 20% of their initial body weight. Animals treated with the RBD mAb COV2-2196 were completely protected from weight loss. COV2-3434 conferred intermediate protection against weight loss (Figure 6B). Pretreatment with COV2-3434 also partially protected against viral burden, with a 7-fold lower level of infectious virus in the lungs compared with the negative control antibody, while we noticed only a minuscule drop in viral load in the nasal turbinates. This might be due to limited access of systemic antibodies to the upper respiratory tract, diffusion barriers, and the lack of active transport receptors (Figure 6C). We repeated the study by passively transferring a higher dose (1 mg/mouse) of COV2-2196 (RBD-specific), COV2-3434 (NTD-specific), or DENV-r2D22 (negative control) mAbs and again observed a comparable reduction of viral titers in the lungs and nasal turbinates (Supplemental Figure 8).

## Discussion

Human neutralizing mAbs against SARS-CoV-2 isolated from recovered COVID-19 individuals are of great importance as potential therapeutic candidates. The continued investigation into identifying protective epitopes using mAbs, as we have done here, may inform a future structure-based rational design of next-generation SARS-CoV-2 vaccines by revealing protective sites whose structure should be preserved in engineered vaccine antigens. Most potently neutralizing SARS-CoV-2 mAbs discovered to date recognize the RBD region, while some moderately neutralizing NTD-directed mAbs were also identified (3–16). All of the NTD-reactive mAbs reported to date have lost their neutralizing capacity against certain emerging VOC. The majority of identified antibodies against NTD target an antigenic site termed the NTD supersite (7, 9–11). Although a few other antigenic sites on NTD have been described, mAbs binding to these sites were generally found to be non-neutralizing. The frequent occurrence of mutations in the NTD of multiple circulating SARS-CoV-2 variants suggests that the NTD is under strong selective pressure from the host’s humoral immune response (38). Furthermore, antigenic changes caused by deletions in NTD have been identified within the antigenic supersite of viruses shed by immunocompromised hosts (39–41).

In this study, we report the isolation and characterization of SARS-CoV-2–neutralizing mAbs targeting the NTD using LIBRA-Seq. We used NTD, a domain cloned from the full-length spike, as antigen bait for isolating memory B cells from a convalescent donor. More than 90% of the clones we selected by LIBRA-Seq for expression reacted exclusively with NTD, and these findings were also supported by reactivity studies with the SARS-CoV-2 S6P_ecto_ domain. We found that a subset of 8 NTD-targeting antibodies selected by LIBRA-Seq was neutralizing. Several of the mAbs potently neutralized VSV-S. The primary target for most of the neutralizing antibodies identified is the NTD supersite, as previously described by several groups (7, 10–12). Most of these NTD supersite–targeting antibodies appear to be members of a public clonotype. Although diverse public clonotypes recognizing RBD or NTD have been described, we identified an *IGHV1-24*–encoded clonotype that seemed to dominate the response to NTD. Clones from this public clonotype are seen following both vaccination and infection.

We also identified an antibody designated COV2-3434 that recognizes a distinct antigenic site on the NTD that may represent a new site of vulnerability on the SARS-CoV-2 S protein. COV2-3434 bound to recombinant SARS-CoV-2 S6P_ecto_ protein weakly in ELISA, but more avidly to cell-surface–displayed S protein on Vero cells infected with VSV-S. In contrast to other NTD-reactive potently neutralizing antibodies, COV2-3434 weakly inhibited infection of VSV-S and authentic SARS-CoV-2 viruses. With these distinctive phenotypes, we tried to learn more about the mode of recognition of this antigenic site by nsEM of antigen-antibody complexes. Unexpectedly, we found that COV2-3434 Fab disrupted SARS-CoV-2 S trimers when added to make S-Fab complexes. This finding of trimer disassociation mediated by COV2-3434 revealed a potential site of vulnerability hidden in the S protein TI. Similarly, a recently identified NTD-reactive neutralizing antibody called 5 to 7 also recognizes a distinct antigenic site within the NTD. Antibodies of this class insert an antibody hypervariable loop into the exposed hydrophobic pocket between the 2 sheets of the NTD β sandwich (42). This pocket was described previously as the binding site for metabolites such as heme with hydrophobic properties (26). Our alanine scan mutagenesis data revealed that COV2-3434 shares some contact residues with the mAb 5 to 7, including F175 and L176, whereas L226 is barely deeper than 175 and 176. However, COV2-3434 also lost its binding capacity when the deep-pocket residue F43 was mutated. We noted that within the S trimer residue F43 lies at an interface between adjacent monomers, such that mAb binding could initiate a destabilization of the trimer.

Recently, several reports about mAbs targeting the TI of multiple viral antigens have been published. For instance, the non-neutralizing influenza mAb FluA20 that recognizes the hemagglutinin TI (43, 44) was identified in an influenza-vaccinated individual. Also, epitope mapping using polyclonal serum from vaccinated rabbits identified antibodies recognizing the HIV envelope glycoprotein TI (45). Similarly, the epitope for a neutralizing mAb against human metapneumovirus (MPV458) lies within the trimeric interface of pneumovirus fusion proteins (46).

COV2-3434 is a rare SARS-CoV-2 S protein TI antibody that mediates virus neutralization. Our COV2-3434 competition data suggest that this class of mAbs may be common in the serum of some vaccinated individuals. Hence, surveillance of this class of antibodies and understanding its contribution to vaccine protection is important, particularly in the context of the emergence of new VOC and updated vaccine designs. Although not all of these TI mAbs neutralize virus in vitro, passive transfer of these mAbs can mitigate severe disease. For example, the FluA20 mAb did not neutralize influenza, but still conferred protection in mice challenged with the H1N1 A/California/04/2009 virus (43). Here, the moderately neutralizing COV2-3434 conferred partial protection against weight loss and lung infection in mice when given as a prophylaxis. We and others in the past have shown that neutralizing NTD or RBD mAbs require Fc effector functions for optimal protection against SARS-CoV-2 infection in vivo (10, 35). Interestingly, a recent study has shown that Fc-mediated effector functions even from a non-neutralizing Ab directed against NTD can contribute to SARS-CoV-2 immunity in combination with an Fc-enhanced mAb by limiting viral spread and infection (47). To this end, it is possible that COV2-3434 could mediate 100% protection in vivo in combination with other NTD or RBD mAbs, a question that should be explored in future studies.

In summary, using LIBRA-Seq, we identified the mAb COV2-3434 that binds to a distinct antigenic site on the NTD and disassociates S trimers by contacting critical residues in a cryptic hydrophobic pocket in the S protein TI.

## Methods

### Resource availability

#### Data availability and material transfer agreements.

All data needed to evaluate the conclusions in the study are present in the manuscript or the supplemental materials. The antibodies in this study are available by material transfer agreement with Vanderbilt University Medical Center. The materials described in this study are available for distribution for nonprofit use using templated documents from the Association of University Technology Managers “Toolkit MTAs” (https://autm.net/surveys-and-tools/agreements/material-transfer-agreements/mta-toolkit). New sequences generated in this study are available in the NCBI’s GenBank under accession numbers ON456498 through ON456513. Sequences for antibodies used as controls in this study were previously submitted to GenBank under accession numbers MT763532 and MT763531.1 (for COV2-2196) and MT665456.1 and MT665069.1 (for COV2-2676).

### Experimental model and details on the study participants

Detailed experimental methods are available in the Supplemental Methods.

### Statistics

The mean ± SEM and the mean ± SD were determined for continuous variables as noted. Technical and biological replicates are described in the figure legends. For analysis of the mouse studies, the comparison of weight change curves was performed by 1-way ANOVA with Dunnett’s post hoc test of the AUC for days 3–6 after infection, using GraphPad Prism version 9.0 (GraphPad Software). Infectious viral loads were compared by 1-way ANOVA with Dunnett’s multiple-comparison test using GraphPad Prism, version 9.0.

### Study approval

#### Study participants.

We studied peripheral blood B cells from 4 individuals with a history of laboratory-confirmed symptomatic SARS-CoV-2 infection. The study was approved by the IRB of Vanderbilt University Medical Center, and specimens were obtained after written informed consent was provided by the participants.

#### Mouse models.

Animal studies were carried out in accordance with the recommendations in the NIH’s *Guide for the Care and Use of Laboratory Animals* (National Academies Press, 2011). The protocols were approved by the IACUC of the Washington University School of Medicine (assurance number A3381–01). Virus inoculations were performed under anesthesia that was induced and maintained with ketamine hydrochloride and xylazine, and all efforts were made to minimize animal suffering. Heterozygous K18-hACE2 c57BL/6J mice (strain 2B6.Cg-Tg(K18-ACE2)2Prlmn/J) were obtained from The Jackson Laboratory (stock no. 034860). Eight- to 9-week-old mice of both sexes were inoculated i.n. with 103 PFU SARS-CoV-2.

## Author contributions

NS and JEC conceived of the project. MSD, ISG, and JEC obtained funding. NS, ARS, REC, EB, LAVB, JBC, KJK, LM, AT, SMD, LSH, RSN, CNS, ECA, CG, and ED performed laboratory experiments. BJD, ISG, RHC, and JEC supervised research. NS and JEC wrote the first draft of the manuscript. All authors reviewed and approved the final manuscript.

## Supplementary Material

Supplemental data

## Version 1 04/26/2022

In-Press Preview

## Version 2 06/01/2022

Electronic publication
