# Supplementary material for: An antibody targeting the N-terminal domain of SARS-CoV-2 disrupts the spike trimer
Source: J Clin Invest. 2022 Jun 1;132(11):e159062. doi: 10.1172/JCI159062 (PMC9151704; doi:10.1172/JCI159062)
Supplement: Supplemental data [file jci-132-159062-s114.pdf]

Supplemental Methods

Cell lines. Vero (ATCC, CCL-81), HEK293 (ATCC, CRL-1573) and HEK293T (ATCC, CRL-3216) cells were maintained at 37°C in 5% CO2 in Dulbecco's minimal essential medium (DMEM) containing 10% (v/v) heat-inactivated fetal bovine serum (FBS), 10 mM HEPES pH 7.3, 1 mM sodium pyruvate, 1× non-essential amino acids and 100 U/mL of penicillin–streptomycin. Vero-furin cells were obtained from T. Pierson (NIAID, NIH) and have been described previously (45) Vero-hACE2-TMPRSS2 cells were a gift of A. Creanga and B. Graham (Vaccine Research Center, NIH). FreeStyle 293F cells (Thermo Fisher Scientific, R79007) were maintained at 37°C in 8% CO2. Expi293F cells (Thermo Fisher Scientific, A1452) were maintained at 37°C in 8% CO2 in Expi293F Expression Medium (Thermo Fisher Scientific, A1435102). ExpiCHO cells (Thermo Fisher Scientific, A29127) were maintained at 37°C in 8% CO2 in ExpiCHO Expression Medium (Thermo Fisher Scientific, A2910002). Mycoplasma testing of Expi293F and ExpiCHO cultures was performed monthly using a PCR-based mycoplasma detection kit (ATCC, 30-1012K).

Antigen purification. A variety of recombinant soluble protein antigens were used in the LIBRA-seq experiment and other experimental assays. For the LIBRA-seq experiment, we used the S6Pecto construct. This plasmid encoded residues 1–1,208 of the SARS-CoV-2 S protein with a mutated S1/S2 cleavage site, proline substitutions at positions 817, 892, 899, 942, 986 and 987, and a C-terminal T4-fibritin trimerization motif, an 8x HisTag, and a TwinStrepTag (SARS-CoV-2 spike HP). DNA encoding this construct was transiently transfected with PEI in Expi293F cells and after six days of expression, supernatants were harvested, and protein was affinity-purified over a StrepTrap HP column (Cytiva Life Sciences). Protein was further resolved to homogeneity over a Superose 6 Increase column (GE Life Sciences).

We generated a plasmid containing a synthesized cDNA encoding a protein designate SARS - CoV-2 S-2P that possessed residues 1–1,208 of the SARS-CoV-2 spike protein as described (1) with a mutated S1/S2 cleavage site, proline substitutions at amino acid positions 986 and 987, a C-terminal T4-fibritin trimerization motif, an 8x HisTag, and a TwinStrepTag. The plasmids were transiently transfected into FreeStyle 293F cells (Thermo Fisher Scientific) using polyethylenimine. The design of the two-proline (2P) forms of the coronavirus trimer spike antigens results in a prefusion-stabilized conformation that better represents neutralization-sensitive epitopes in comparison to their wild-type forms. Two h after transfection, cells were treated with kifunensine to ensure uniform glycosylation. Transfected supernatants were harvested after 6 days of expression. SARS-CoV-2 S1 (cat. no: 40591-V08B1), SARS-CoV-2 S2 (cat. no: 40590-V08B), SARS-CoV-2 RBD (cat. no: 40592-V05H) and SARS-CoV-2 NTD (cat. no: 40591-V41H-B-20) truncated proteins were purchased (Sino Biological).

A gene encoding the ectodomain of a pre-fusion conformation-stabilized SARS-CoV-2 S protein ectodomain (S6Pecto) (2) was synthesized and cloned into a DNA plasmid expression vector for mammalian cells. A similarly designed S protein antigen with two prolines and removal of the furin cleavage site for stabilization of the prefusion form of S (S2Pecto) was reported previously (1). In brief, this gene includes the ectodomain of SARS-CoV-2 (to residue 1,208), a T4 fibritin trimerization domain, an AviTag site-specific biotinylation sequence and a C-terminal 8× His tag. To stabilize the construct in the pre-fusion conformation, we included substitutions F817P, A892P, A899P, A942P, K986P and V987P and mutated the furin cleavage site at residues 682–685 from RRAR to ASVG. The recombinant S6Pecto protein was isolated by metal affinity chromatography on HisTrap Excel columns (Cytiva), and protein preparations were purified further by size-exclusion chromatography on a Superose 6 Increase 10/300 column (Cytiva). The presence of

trimeric, pre-fusion conformation S protein was verified by negative-stain electron microscopy (3). For electron microscopy with S protein and Fabs, we expressed a variant of S6Pecto lacking an AviTag but containing a C-terminal Twin-Strep-tag, similar to that described previously (3). Expressed protein was isolated by metal affinity chromatography on HisTrap Excel columns (Cytiva), followed by further purification on a StrepTrap HP column (Cytiva) and size-exclusion chromatography on TSKgel G4000SWXL (TOSOH).

DNA-barcoding of antigens:

We used oligonucleotides that possess a 15-basepair antigen barcode, a sequence capable of annealing to the template-switch oligonucleotide that is part of the 10X Genomics bead-delivered oligonucleotides and contain truncated TruSeq small RNA read-1 sequences in the following structure: 5'-CCTTGGCACCCGAGAATTCCANNNNNNNNNNNNNCCCATATAAGA*A*A-3', where Ns represent the antigen barcode as previously described (4). For each antigen, a unique DNA barcode was directly conjugated to the antigen itself. In particular, 5'amino-oligonucleotides were conjugated directly to each antigen using the SoluLINK Protein-Oligonucleotide Conjugation Kit (TriLink cat. no. S-9011) according to manufacturer's instructions. Briefly, the oligonucleotide and protein were desalted, and then the amino-oligo was modified with the 4FB crosslinker, and the biotinylated antigen protein was modified with S-HyNic. Then, the 4FB-oligo and the HyNic-antigen were mixed. This action causes a stable bond to form between the protein and the oligonucleotide. The concentration of the antigen-oligo conjugates was determined by a BCA assay, and the HyNic molar substitution ratio of the antigen-oligo conjugates was analyzed using a NanoDrop instrument according to the SoluLINK protocol guidelines. Chromatography separation on an AKTA FPLC instrument was used to remove excess oligonucleotide from the

protein-oligo conjugates, which were also verified using SDS-PAGE with a silver stain. Antigen-oligo conjugates also were used in flow cytometry titration experiments.

METHOD DETAILS

Antigen-specific B cell sorting. Cells were stained and mixed with DNA-barcoded antigens and other antibodies, and then sorted using fluorescence activated cell sorting (FACS). First, cells were counted, and viability was assessed using Trypan Blue. Then, cells were washed three times with DPBS supplemented with 0.1% bovine serum albumin (BSA). Cells were resuspended in DPBS-BSA and stained with cell markers including viability dye (Ghost Red 780), CD14-APC-Cy7, CD3-FITC, CD19-BV711, and IgG-PE-Cy5. Additionally, antigen-oligo conjugates were added to the stain. After staining in the dark for 30 min at room temperature, cells were washed three times with DPBS-BSA at 300 x g for five min. Cells then were incubated for 15 min at room temperature with Streptavidin-PE to label cells with bound antigen. Cells were washed three times with DPBS-BSA, resuspended in DPBS, and sorted by FACS. Antigen-positive cells were bulk sorted and delivered to the Vanderbilt Technologies for Advanced Genomics (VANTAGE) sequencing core laboratory at an appropriate target concentration for 10X Genomics library preparation and subsequent sequence analysis. FACS data were analyzed using FlowJo™ Software (Mac) version 10.6 (Becton, Dickinson).

Sample preparation, library preparation, and sequencing. Single-cell suspensions were loaded onto a Chromium Controller microfluidics device (10X Genomics) and processed using the B-cell Single Cell V(D)J solution according to manufacturer's suggestions for a target capture of 10,000 B cells per 1/8 10X cassette, with minor modifications to intercept, amplify and purify the antigen barcode libraries as previously described (4).

Sequence processing and bioinformatic analysis. We used our previously described pipeline to use paired-end FASTQ files of oligo libraries as input, process and annotate reads for cell barcode, UMI, and antigen barcode, and generate a cell barcode - antigen barcode UMI count matrix ((4, 5). BCR contigs were processed using Cell Ranger software (10X Genomics) using GRCh38 as reference. Antigen barcode libraries were also processed using Cell Ranger. The overlapping cell barcodes between the two libraries were used as the basis of the subsequent analysis. We removed cell barcodes that had only non-functional heavy chain sequences and cells with multiple functional heavy chain sequences and/or multiple functional light chain sequences, reasoning that these may be multiplets. Additionally, we aligned the BCR contigs (filtered_contigs.fasta file output by Cell Ranger, 10X Genomics) to IMGT reference genes using HighV-Quest (6). The output of HighV-Quest was parsed using ChangeO (7) and merged with an antigen barcode UMI count matrix. Finally, we determined the LIBRA-seq score for each antigen in the library for every cell as previously described (4).

High-throughput antibody expression. For high-throughput production of recombinant antibodies, approaches were used that are designated as microscale. For antibody expression, microscale transfections were performed (~1 mL per antibody) of Chinese hamster ovary (CHO) cell cultures using the Gibco ExpiCHO Expression System and a protocol for deep 96-well blocks (Thermo Fisher Scientific). In brief, synthesized antibody-encoding DNA (~2 μg per transfection) was added to OptiPro serum free medium (OptiPro SFM), incubated with ExpiFectamine CHO Reagent and added to 800 µL of ExpiCHO cell cultures into 96-deep-well blocks using a ViaFlo 384 liquid handler (Integra Biosciences). The plates were incubated on an orbital shaker at 1,000 r.p.m. with an orbital diameter of 3 mm at 37°C in 8% CO2. The day after transfection,

ExpiFectamine CHO Enhancer and ExpiCHO Feed reagents (Thermo Fisher Scientific) were added to the cells, followed by 4 d incubation for a total of 5 d at 37°C in 8% CO2. Culture supernatants were collected after centrifuging the blocks at 450 x g for 5 min and were stored at 4°C until use. For high-throughput microscale antibody purification, fritted deep-well plates were used containing 25 μL of settled protein G resin (GE Healthcare Life Sciences) per well. Clarified culture supernatants were incubated with protein G resin for antibody capturing, washed with PBS using a 96-well plate manifold base (Qiagen) connected to the vacuum and eluted into 96-well PCR plates using 86 μL of 0.1 M glycine-HCL buffer pH 2.7. After neutralization with 14 μL of 1 M Tris-HCl pH 8.0, purified antibodies were buffer-exchanged into PBS using Zeba Spin Desalting Plates (Thermo Fisher Scientific) and stored at 4°C until use.

MAb production and purification. cDNAs encoding mAbs of interest were synthesized (Twist Bioscience) and cloned into an IgG1 monocistronic expression vector (designated as pTwist-mCis_G1) or Fab expression vector (designated as pTwist-mCis_FAB) and used for production in mammalian cell culture. This vector contains an enhanced 2A sequence and GSG linker that allows for the simultaneous expression of mAb heavy and light chain genes from a single construct upon transfection (8). For antibody production, we performed transfection of ExpiCHO cell cultures using the Gibco ExpiCHO Expression System as described by the vendor. IgG molecules were purified from culture supernatants using HiTrap MabSelect SuRe (Cytiva) on a 24-column parallel protein chromatography system (Protein BioSolutions).

Fab proteins were purified using CaptureSelect column (Thermo Fisher Scientific). Purified antibodies were buffer-exchanged into PBS, concentrated using Amicon Ultra-4 50-kDa (IgG) or 30 kDa (Fab) centrifugal filter units (Millipore Sigma) and stored at 4°C until use. F(ab¢)2

fragments were generated after cleavage of IgG with IdeS protease (Promega) and then purified using TALON metal affinity resin (Takara) to remove the enzyme and protein A agarose (Pierce) to remove the Fc fragment. Purified mAbs were tested routinely for endotoxin levels and found to be less than 30 EU per mg IgG. Endotoxin testing was performed using the PTS201F cartridge (Charles River), with a sensitivity range from 10 to 0.1 EU per mL, and an Endosafe Nexgen-MCS instrument (Charles River).

Focus reduction neutralization test (FRNT). Serial dilutions of serum/plasma were incubated with 102 FFU of SARS-CoV-2 for 1 h at 37°C. The antibody-virus complexes were added to Vero E6 cell-culture monolayers in 96-well plates for 1 h at 37°C. Cells then were overlaid with 1% (w/v) methylcellulose in minimum essential medium (MEM) supplemented to contain 2% heat-inactivated FBS. Plates were fixed 30 h later by removing overlays and fixed with 4% paraformaldehyde (PFA) in PBS for 20 min at room temperature. The plates were incubated sequentially with 1 μg/mL of rCR3022 anti-S antibody or a murine anti-SARS-CoV-2 mAb, SARS2-16 (hybridoma supernatant diluted 1:6,000 to a final concentration of ~20 ng/mL) and then HRP-conjugated goat anti-human IgG (Sigma-Aldrich, A6029) in PBS supplemented with 0.1% (w/v) saponin (Sigma) and 0.1% BSA. SARS-CoV-2-infected cell foci were visualized using TrueBlue peroxidase substrate (KPL) and quantitated on an ImmunoSpot 5.0.37 Macro Analyzer (Cellular Technologies). Half maximal inhibitory concentration (IC_{50}) values were determined by nonlinear regression analysis (with a variable slope) using Prism software.

Conventional throughput neutralization assay. To determine neutralizing activity of serum/plasma and IgG, we used real-time cell analysis (RTCA) assay on an xCELLigence RTCA MP Analyzer (ACEA Biosciences Inc.) that measures virus-induced cytopathic effect (CPE) (3, 9). Briefly, 50 μL of cell culture medium (DMEM supplemented with 2% FBS) was added to each

well of a 96-well E-plate using a ViaFlo384 liquid handler (Integra Biosciences) to obtain background reading. A suspension of 18,000 Vero-E6 cells in 50 µL of cell culture medium was seeded in each well, and the plate was placed on the analyzer. Measurements were taken automatically every 15 min, and the sensograms were visualized using RTCA software version 2.1.0 (ACEA Biosciences Inc). VSV-S (0.01 MOI, ~120 PFU per well) was mixed 1:1 with a dilution of serum/plasma or mAb in a total volume of 100 µL using DMEM supplemented with 2% FBS as a diluent and incubated for 1 h at 37°C in 5% CO2. At 16 h after seeding the cells, the virus-mAb mixtures were added in replicates to the cells in 96-well E-plates. For the biliverdin assay, biliverdin was added to the virus at a final concentration of 25 µM before addition to the antibody; similarly, polysorbate-80 was added to the virus at 0.02% before addition to the antibody. Triplicate wells containing virus only (maximal CPE in the absence of mAb) and wells containing only Vero cells in medium (no-CPE wells) were included as controls. Plates were measured continuously (every 15 min) for 48 h to assess virus neutralization. Normalized cellular index (CI) values at the endpoint (48 h after incubation with the virus) were determined using the RTCA software version 2.1.0 (ACEA Biosciences Inc.). Results are expressed as percent neutralization in a presence of respective mAb relative to control wells with no CPE minus CI values from control wells with maximum CPE. RTCA IC50 values were determined by nonlinear regression analysis using Prism software.

Electron microscopy sample and grid preparation, imaging and processing of S6Pecto–Fab complexes. For electron microscopy imaging of spike protein and Fabs, we expressed a variant of S6Pecto containing a C-terminal Twin-Strep-tag, similar to that described previously (3). Expressed protein was incubate with BioLock (IBA Lifesciences) and then isolated by Strep affinity chromatography on StrepTrap HP columns (GE Healthcare). Fabs were expressed as a

recombinant Fab and purify with affinity column. For screening and imaging of negatively-stained SARS-CoV-2 S6Pecto protein in complex with human Fabs, the proteins were incubated at a Fab:spike molar ratio of 4:1 for about 1 hour at ambient temperature or overnight at 4°C, and approximately 3 μL of the sample at concentrations of about 10 to 15 μg/mL was applied to a glow-discharged grid with continuous carbon film on 400 square mesh copper electron microscopy grids (Electron Microscopy Sciences). The grids were stained with 0.75% uranyl formate (10). Images were recorded on a Gatan US4000 4k×4k CCD camera using an FEI TF20 (TFS) transmission electron microscope operated at 200 keV and control with Serial EM (11). All images were taken at 50,000× magnification with a pixel size of 2.18 Å per pixel in low-dose mode at a defocus of 1.5–1.8 μm. The total dose for the micrographs was around 30e−per Å2. Image processing was performed using the cryoSPARC (12) software package. Images were imported, CTF-estimated and particles were picked. The particles were extracted with a box size of 256 pixels and binned to 128 pixels (pixel size of 4.36 A/pix) and 2D class averages were performed (see also Supplementary Table 3 for detailed). For time point of the complex with Fab Cov2-3434, SARS-CoV-2 S6Pecto protein and the Fab was mixed at ambient temperature and samples of ~3 μL were pulled at the time points and applied to the grid and stained.

Serum antibody competition binding ELISAs with biotinylated reference mAbs. mAb COV2-3434 was biotinylated using NHS-PEG4-biotin (Thermo Fisher Scientific, cat# A39259) according to manufacturer protocol. Following biotinylation, biotinylated COV2-3434 was titrated in ELISA to verify specific binding and verify if EC50 was similar to the un-biotinylated antibody. Serum samples for use in competition ELISA were heat inactivated by incubation at 55°C for 1 hr. ELISAs were performed using 384-well plates that were coated overnight at 1 μg/mL with S6Pecto containing a C-terminal Twin-Strep-tag, similar to that described previously (3). The following day, plates were washed three times with PBS-T and blocked with 2% bovine serum

albumin (BSA) in PBS containing 0.05% Tween-20 (blocking buffer). Plates were washed three times with PBS-T and two-fold serial dilutions of donor serum (1:10 initial dilution) or control mAb (20,000 ng/mL initial dilution) in blocking buffer were added to each plate (total volume 25 µL/well) and incubated at RT for 1 hr. After incubation, 5 µL of biotinylated COV2-3434 (20 µg/mL) in blocking buffer were added directly to the wells containing the serial dilutions of competing serum or COV2-3434 mAb. The concentration of biotinylated mAb was calculated to be at approximately the EC90 of the mAb after addition to an equal volume of competing serum or mAb in the plate. Plates were incubated for 30 min at RT and then washed three times with PBS-T. After this wash, HRP-conjugated avidin (Sigma Aldrich, 1:3,500 dilution) in blocking buffer was added and plates were incubated for 1 h. After incubation, plates were washed three times with PBS-T and 25 µL of a 3,3′,5,5′- tetramethylbenzidine (TMB) substrate (Thermo Fisher Scientific) was added to each well. After sufficient development, the reaction was quenched by addition of 25 µL 1 M HCl and the optical density values were measured at 450 nm wavelength on a BioTek plate reader. For each plate, background signal (signal from wells that were not coated with antigen) was subtracted and values were normalized to no-competition controls (signal from wells that had no competing serum or mAb) Four-parameter dose-response/inhibition curves were fit to the normalized data using Prism software (GraphPad) v8.1.1. Each dilution of serum or mAb was performed in triplicate and each experiment was conducted at least twice independently.

Epitope mapping of antibodies by alanine-scanning mutagenesis. Epitope mapping was performed essentially as described previously (13) using a SARS-CoV-2 (strain Wuhan-Hu-1) spike protein NTD shotgun mutagenesis mutation library, made using a full-length expression construct for spike protein, where 215 residues of the NTD (between spike residues 9 and 310) were mutated individually to alanine, and alanine residues to serine. Mutations were confirmed by

DNA sequencing, and clones arrayed in a 384-well plate, one mutant per well. Binding of mAbs to each mutant clone in the alanine scanning library was determined, in duplicate, by high-throughput flow cytometry. A plasmid encoding cDNA for each spike protein mutant was transfected into HEK-293T cells and allowed to express for 22 h. Cells were fixed in 4% (v/v) paraformaldehyde (Electron Microscopy Sciences), and permeabilized with 0.1% (w/v) saponin (Sigma-Aldrich) in PBS plus calcium and magnesium (PBS++) before incubation with mAbs diluted in PBS++, 10% normal goat serum (Sigma), and 0.1% saponin. MAb screening concentrations were determined using an independent immunofluorescence titration curve against cells expressing wild-type S protein to ensure that signals were within the linear range of detection. Antibodies were detected using 3.75 μg/mL of Alexa-Fluor-488-conjugated secondary antibodies (Jackson ImmunoResearch Laboratories) in 10% normal goat serum with 0.1% saponin. Cells were washed three times with PBS++/0.1% saponin followed by two washes in PBS, and mean cellular fluorescence was detected using a high-throughput Intellicyte iQue flow cytometer (Sartorius). Antibody reactivity against each mutant S protein clone was calculated relative to wild-type S protein reactivity by subtracting the signal from mock-transfected controls and normalizing to the signal from wild-type S-transfected controls. Mutations within clones were identified as critical to the mAb epitope if they did not support reactivity of the test MAb but supported reactivity of other SARS-CoV-2 antibodies. This counter-screen strategy facilitates the exclusion of S protein mutants that are locally misfolded or have an expression defect.

Measurement of viral burden. Plaque assays were performed as described previously (14, 15) on Vero+TMPRSS2+hACE2 cells. Briefly, lung homogenates were serially diluted and added to Vero+TMPRSS2+hACE2 cell monolayers in 12-well plates. Plates were incubated at 37 °C for 1 h and then overlaid with 1% (w/v) methylcellulose in MEM supplemented with 2% FBS. Plates

were incubated at 37 °C for 72 h and were then fixed with 4% PFA for 20 min. Plaques were visualized by staining with 0.05% crystal violet in 20% methanol.

ELISA binding assays. Wells of 96-well microtiter plates were coated with purified recombinant SARS-CoV-2 S6Pecto, SARS-CoV-2 S NTD, or SARS-CoV-2 RBS protein at 4 °C overnight. Plates were blocked with 2% non-fat dry milk and 2% normal goat serum in Dulbecco's phosphate-buffered saline (DPBS) containing 0.05% Tween-20 (DPBS-T) for 1 h. The bound antibodies were detected using goat anti-human IgG conjugated with horseradish peroxidase (HRP) (Southern Biotech, cat. 2040-05, lot B3919-XD29, 1:5,000 dilution) and a 3,3′,5,5′-tetramethylbenzidine (TMB) substrate (Thermo Fisher Scientific). Color development was monitored, 1 M HCl was added to stop the reaction, and the absorbance was measured at 450 nm using a spectrophotometer (Biotek). For dose–response assays, serial dilutions of purified mAbs were applied to the wells in triplicate, and antibody binding was detected as detailed above. Half maximal effective concentration (EC_{50}) values for binding were determined using Prism v.8.0 software (GraphPad) after log transformation of the mAb concentration using sigmoidal dose–response nonlinear regression analysis.

Human ACE2 binding inhibition analysis. Wells of 384-well microtiter plates were coated with 1 µg/mL purified recombinant SARS-CoV-2 S6Pecto protein at 4°C overnight. Plates were blocked with 2% non-fat dry milk and 2% normal goat serum in DPBS-T for 1 h. For screening assays, mAbs were diluted two-fold in blocking buffer starting from 20 µg/mL in triplicate, added to the wells (20 µL per well) and incubated for 1 h at ambient temperature. Recombinant human ACE2 with a C-terminal Flag tag peptide was added to wells at 2 µg/mL in a 5 µL per well volume (final 0.4 µg/mL concentration of human ACE2) without washing of antibody and then incubated for 40 min at ambient temperature. Plates were washed and bound human ACE2 was detected

using HRP-conjugated anti-Flag antibody (Sigma-Aldrich, cat. A8592, lot SLBV3799, 1:5,000 dilution) and TMB substrate. ACE2 binding without antibody served as a control. The signal obtained for binding of the human ACE2 in the presence of each dilution of tested antibody was expressed as a percentage of the human ACE2 binding without antibody after subtracting the background signal. For dose–response assays, serial dilutions of purified mAbs were applied to the wells in triplicate, and mAb binding was detected as detailed above. IC50 values for inhibition by mAb of S6Pecto protein binding to human ACE2 was determined after log transformation of antibody concentration using sigmoidal dose–response nonlinear regression analysis.

Cell-surface antigen-display assay. Vero cell monolayers were monitored until 80% confluent and then inoculated with VSV-SARS-CoV-2 virus (Wa1/2020 strain) (designated here as VSV-S) at an MOI of 0.5 in culture medium (DMEM with 2% FBS). For a T-225 flask, 10 mL of diluted VSV-S virus was added to the monolayer, then incubated for 40 min. During the incubation, the flask was gently rocked back and forth every 10 min to ensure even infection. Following, the incubation the flask volume was topped off to 30 mL with 2% FBS containing DMEM and incubated for 14 h. Cells were monitored for CPE under a microscope, were trypsinized and washed in FACS buffer. 100,000 infected cells were seeded per well to stain with respective antibodies. All antibody was diluted to 10 µg/mL in FACS buffer, and then serially diluted 3-fold 7 times to stain for antibodies that react to cell-surface-displayed S protein. Infected cells then were resuspended in 50 µL of diluted antibody. Antibody binding was detected with anti-IgG Alexa-Fluor-647-labelled secondary antibodies. Cells were analyzed on an iQue cytometer for staining first by gating to identify infected cells as indicated by GFP-positive cells, and then gated for secondary antibody binding.

Cell-surface antibody-mediated S1 shedding. CHO-K1 cells stably expressing the prototypic SARS-CoV-2 spike protein were collected, washed in wash buffer (PBS, 1% BSA, 2 mM EDTA) and resuspended in PBS. Hundred-thousand cells per well were dispensed into round bottom 96-well plates (Corning), and treated with 10 µg/mL TPCK-Trypsin (Sigma-Aldrich Cat.no: T1426-50MG) for 30 min at 37 °C. Cells were washed and incubated with 15 µg/mL antibody across 5, 30, 60, 120 and 180 min time points at 37 °C. Cells were washed with ice-cold wash buffer, and stained with PE-conjugated goat anti-human IgG secondary antibody (southern biotech cat.no:2014-09) for 30 min on ice in the dark. Cells were washed twice with cold wash buffer and analyzed using iQue. Binding at each time point was measured as median fluorescence intensity (MFI) in triplicates for each mAb and relative mAb binding was calculated. Data were analyzed and plotted using GraphPad Prism v. 9.0.1.

High-throughput real-time cell analysis (RTCA) neutralization assay. To screen for neutralizing activity in the panel of recombinantly expressed mAbs, we used a high-throughput and quantitative RTCA assay and xCelligence RTCA HT Analyzer (ACEA Biosciences) that assesses kinetic changes in cell physiology, including virus-induced cytopathic effect (CPE). Twenty µL of cell culture medium (DMEM supplemented with 2% FBS) was added to each well of a 384-well E-plate using a ViaFlo384 liquid handler (Integra Biosciences) to obtain background reading. Six thousand (6,000) Vero-furin cells in 20 µL of cell culture medium were seeded per well, and the plate was placed on the analyzer. Sensograms were visualized using RTCA HT software version 1.0.1 (ACEA Biosciences). For a screening neutralization assay, equal amounts of virus were mixed with micro-scale purified antibodies in a total volume of 40 µL using DMEM supplemented with 2% FBS as a diluent and incubated for 1 h at 37°C in 5% CO2. At ~17–20 h after seeding the cells, the virus–mAb mixtures were added to the cells in 384-well E-plates. Wells

containing virus only (in the absence of mAb) and wells containing only Vero cells in medium were included as controls. Plates were measured every 8–12 h for 48–72 h to assess virus neutralization. Micro-scale antibodies were assessed in four 5-fold dilutions (starting from a 1:20 sample dilution), and their concentrations were not normalized. In some experiments, mAbs were tested in triplicate using a single (1:20) dilution. Neutralization was calculated as the percent of maximal cell index in control wells without virus minus cell index in control (virus-only) wells that exhibited maximal CPE at 40 to 48 h after applying virus–antibody mixture to the cells. A mAb was classified as fully neutralizing if it completely inhibited SARS-CoV-2-induced CPE at the highest tested concentration, while a mAb was classified as partially neutralizing if it delayed but did not fully prevent CPE at the highest tested concentration.

Protection against SARS-CoV-2 in mice. Animal studies were carried out in accordance with the recommendations in the Guide for the Care and Use of Laboratory Animals of the National Institutes of Health. The protocols were approved by the Institutional Animal Care and Use Committee at the Washington University School of Medicine (Assurance number A3381-01). Virus inoculations were performed under anesthesia that was induced and maintained with ketamine hydrochloride and xylazine, and all efforts were made to minimize animal suffering.

Female heterozygous K18-hACE C57BL/6J mice were housed in groups of up to 5 mice per cage at 18 to 24°C ambient temperatures and 40 to 60% humidity. Mice were fed a 20% protein diet (PicoLab 5053, Purina) and maintained on a 12-h light–dark cycle (06:00 to 18:00). Food and water were available ad libitum. Mice (8 to 9 weeks old) were inoculated with 1×10^4 focus forming units of SARS-CoV-2 (viral titer was determined on Vero-TMPRSS2-ACE2 cells) via the intranasal route. Anti-SARS-CoV-2 human mAbs or isotype control mAbs were administered 24

h before (prophylaxis) SARS-CoV-2 inoculation. Weights and lethality were monitored daily for up to 6 days after inoculation and mice were euthanized at 6 dpi and tissues were collected.

References:

1. Wrapp D, Wang N, Corbett KS, Goldsmith JA, Hsieh CL, Abiona O, et al. Cryo-EM structure of the 2019-nCoV spike in the prefusion conformation. *Science.* 2020;367(6483):1260-3.
2. Hsieh CL, Goldsmith JA, Schaub JM, DiVenere AM, Kuo HC, Javanmardi K, et al. Structure-based design of prefusion-stabilized SARS-CoV-2 spikes. *Science.* 2020;369(6510):1501-5.
3. Zost SJ, Gilchuk P, Chen RE, Case JB, Reidy JX, Trivette A, et al. Rapid isolation and profiling of a diverse panel of human monoclonal antibodies targeting the SARS-CoV-2 spike protein. *Nat Med.* 2020;26(9):1422-7.
4. Setliff I, Shiakolas AR, Pilewski KA, Murji AA, Mapengo RE, Janowska K, et al. High-Throughput Mapping of B Cell Receptor Sequences to Antigen Specificity. *Cell.* 2019;179(7):1636-46 e15.
5. Shiakolas AR, Kramer KJ, Wrapp D, Richardson SI, Schafer A, Wall S, et al. Cross-reactive coronavirus antibodies with diverse epitope specificities and Fc effector functions. *Cell Rep Med.* 2021;2(6):100313.
6. Alamyar E, Duroux P, Lefranc MP, and Giudicelli V. IMGT((R)) tools for the nucleotide analysis of immunoglobulin (IG) and T cell receptor (TR) V-(D)-J repertoires, polymorphisms, and IG mutations: IMGT/V-QUEST and IMGT/HighV-QUEST for NGS. *Methods Mol Biol.* 2012;882:569-604.

7. Gupta NT, Vander Heiden JA, Uduman M, Gadala-Maria D, Yaari G, and Kleinstein SH. Change-O: a toolkit for analyzing large-scale B cell immunoglobulin repertoire sequencing data. *Bioinformatics.* 2015;31(20):3356-8.

8. Chng J, Wang T, Nian R, Lau A, Hoi KM, Ho SC, et al. Cleavage efficient 2A peptides for high level monoclonal antibody expression in CHO cells. *MAbs.* 2015;7(2):403-12.

9. Gilchuk P, Bombardi RG, Erasmus JH, Tan Q, Nargi R, Soto C, et al. Integrated pipeline for the accelerated discovery of antiviral antibody therapeutics. *Nat Biomed Eng.* 2020;4(11):1030-43.

10. Ohi M, Li Y, Cheng Y, and Walz T. Negative Staining and Image Classification - Powerful Tools in Modern Electron Microscopy. *Biol Proced Online.* 2004;6:23-34.

11. Mastronarde DN. Automated electron microscope tomography using robust prediction of specimen movements. *Journal of Structural Biology.* 2005;152(1):36-51.

12. Punjani A, Rubinstein JL, Fleet DJ, and Brubaker MA. cryoSPARC: algorithms for rapid unsupervised cryo-EM structure determination. *Nature Methods.* 2017;14(3):290-+.

13. Davidson E, and Doranz BJ. A high-throughput shotgun mutagenesis approach to mapping B-cell antibody epitopes. *Immunology.* 2014;143(1):13-20.

14. Hassan AO, Case JB, Winkler ES, Thackray LB, Kafai NM, Bailey AL, et al. A SARS-CoV-2 Infection Model in Mice Demonstrates Protection by Neutralizing Antibodies. *Cell.* 2020;182(3):744-53 e4.

15. Case JB, Rothlauf PW, Chen RE, Liu Z, Zhao H, Kim AS, et al. Neutralizing Antibody and Soluble ACE2 Inhibition of a Replication-Competent VSV-SARS-CoV-2 and a Clinical Isolate of SARS-CoV-2. *Cell Host Microbe.* 2020;28(3):475-85 e5.

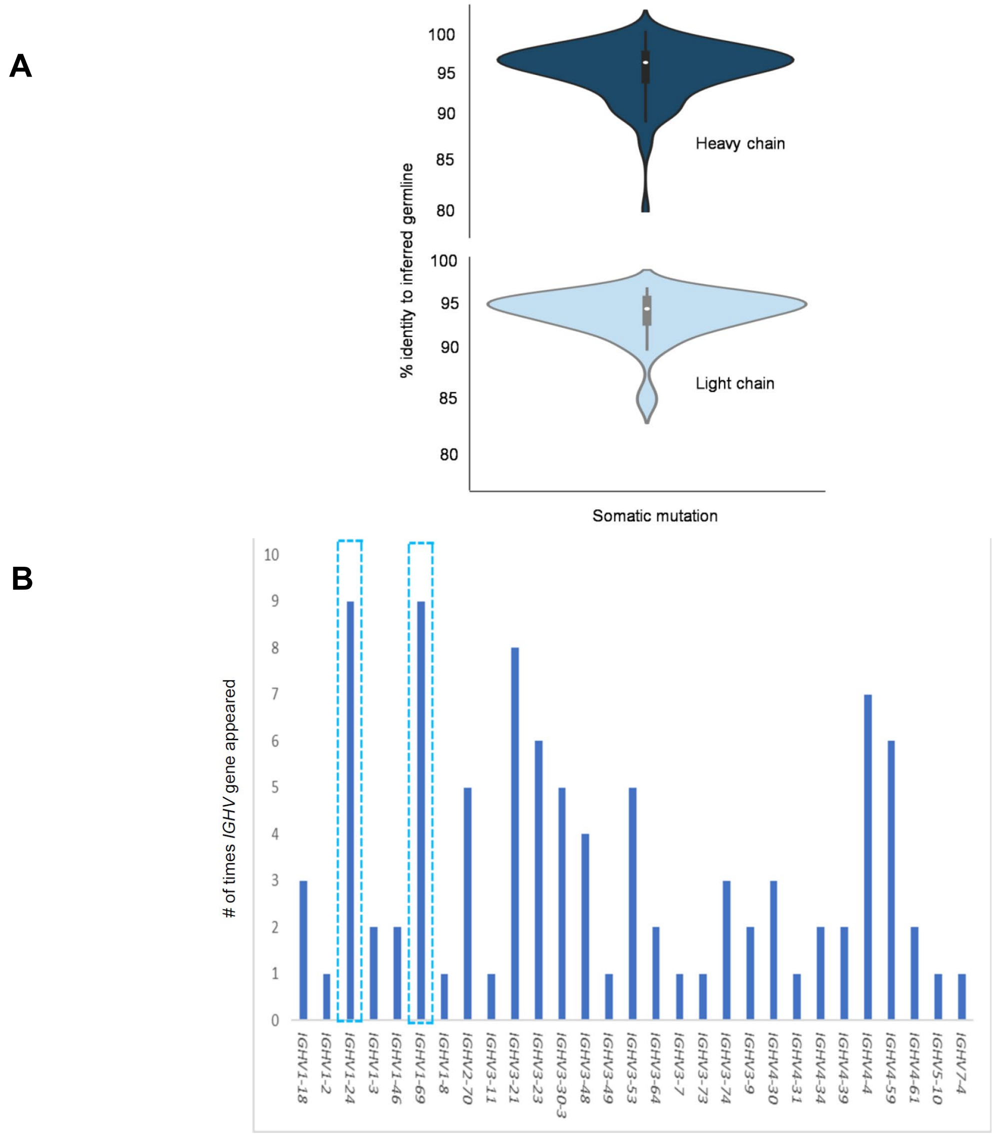

Figure S1. Divergence from inferred germline gene sequences, related to Figure 2

A. The number of mutations of each mAb relative to the inferred germline variable gene was counted for each clone. These numbers then were transformed into percent values and plotted as violin plots. For the heavy chain, values range from 80.4 to 100, with a median of 96.6, a 25th quartile of 94.6 and a 75th quartile of 97.6. For the light chain, values range from 88.1 to 100, with a median of 97.9, a 25th quartile of 96.5 and a 75th quartile of 98.9.
B. Bar graph showing IGHV gene usage by 102 clones expressed Y-axis represents number of times same IGHV appeared and on x-axis is the IGHV gene identified.

A

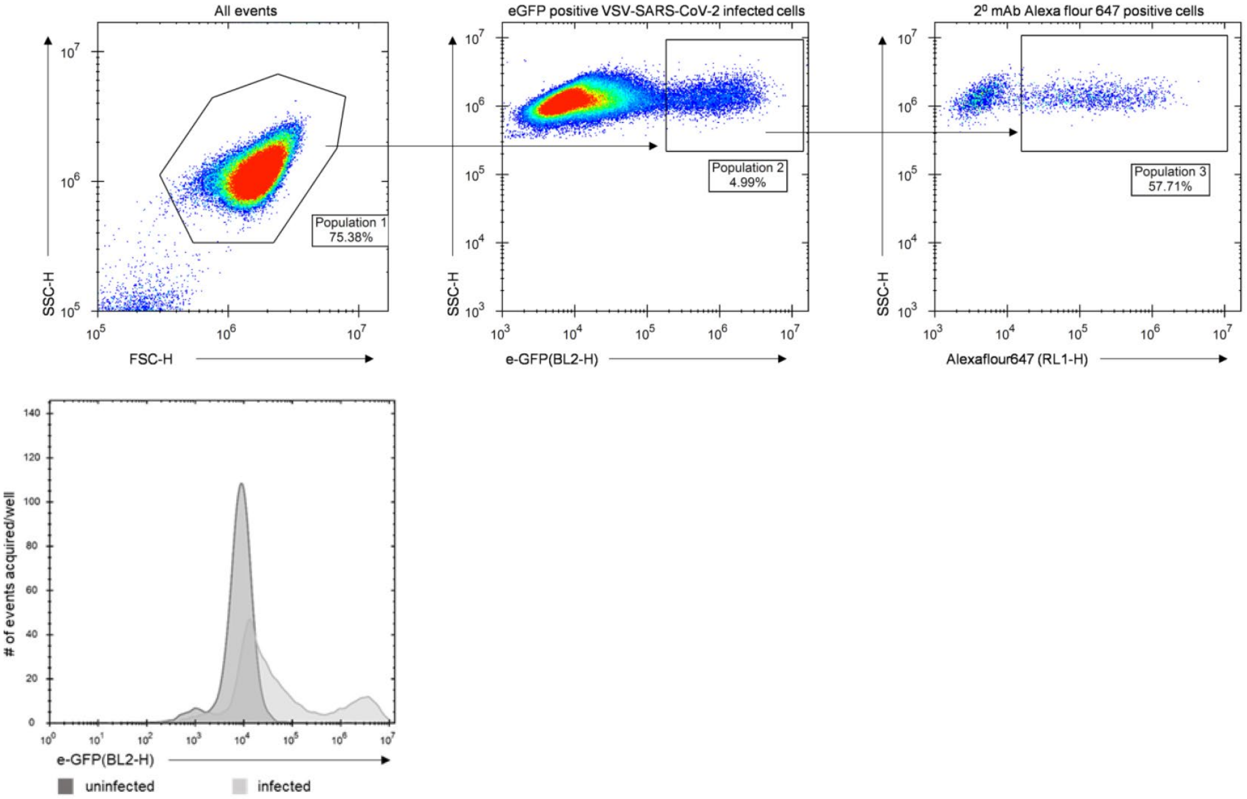

B

Figure S2. Gating strategy used for cell-surface antigen-display experiment, related to Figure 3

A. The first gate is for all cells, the second gate is for infected cells, and the third gate is for antibody binding to infected cells.

B. Overlay of histograms infected cells in light grey on uninfected cells in dark grey gated for Alexa Fluor 647 staining.

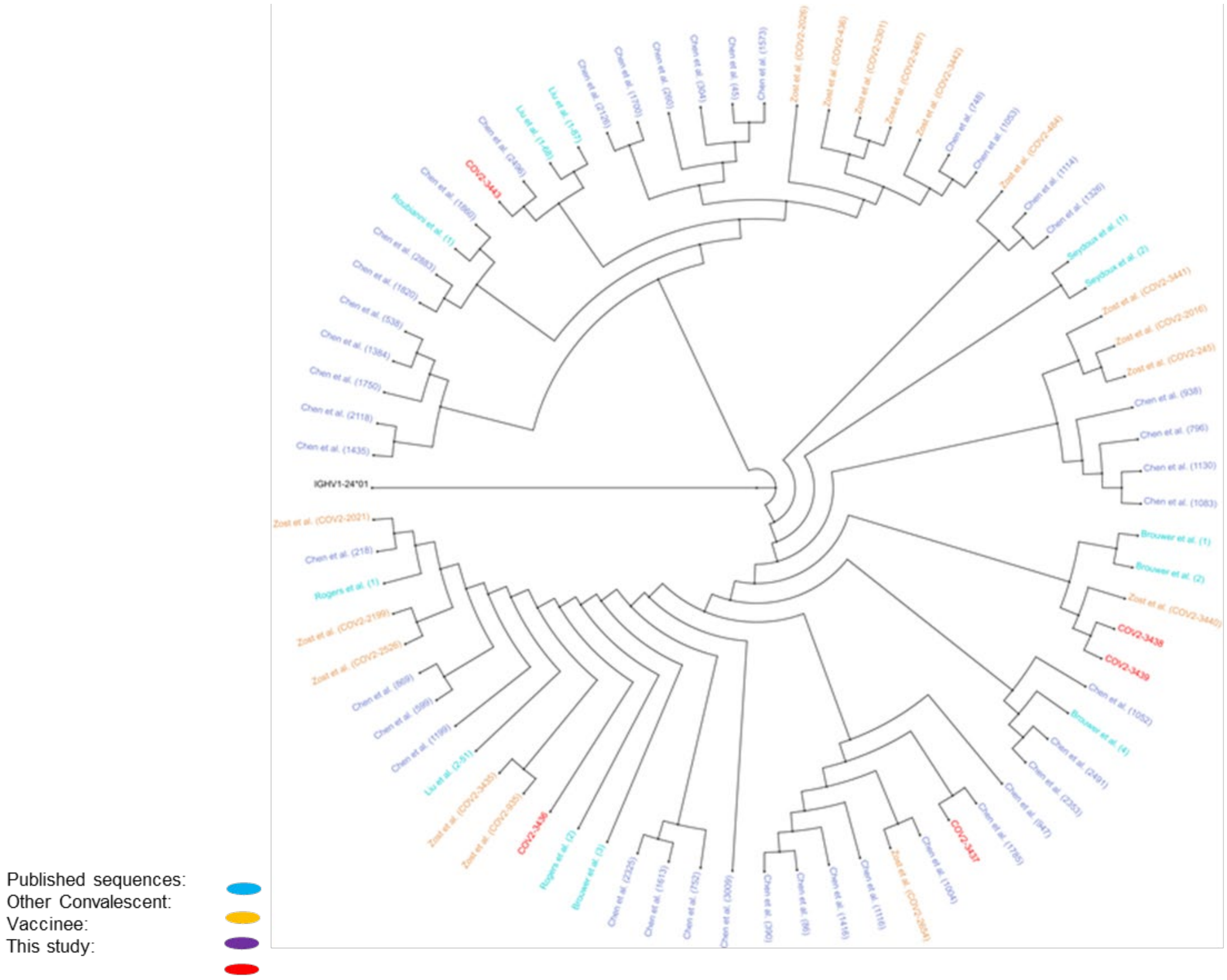

Figure S3. **Phylogenetic tree obtained after aligning multiple sequence of the heavy chain of *IGHV1-24* genes, related to Figure 3** identified in this study (red) with IGHV 1-24 genes available from sequences 1) deposited in public databases shown in cyan color, 2) from vaccinated individuals shown in purple, or 3) from infected individuals shown in orange.

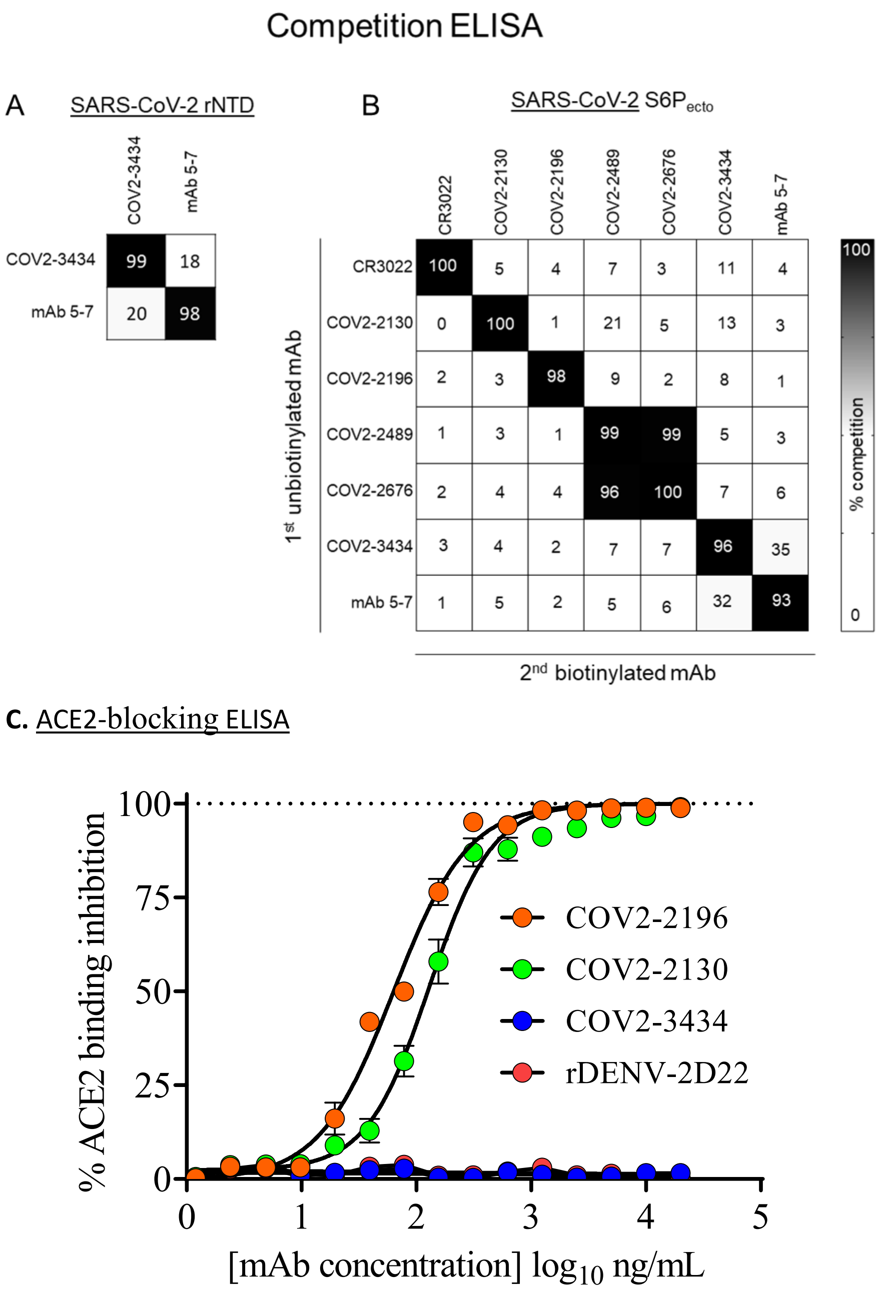

Figure S4. Competition ELISA of mAbs, related to Figure 4

Competition ELISA of mAbs with previously mapped antibodies COV2-2130, COV2-2196, COV2-2676, COV2-2489, r4A8 or rCR3022. Unlabeled antibodies applied to antigen first are indicated on the left, while biotinylated antibodies that were added to antigen-coated wells second are listed across the top. The number in each box represents the percent competition binding of the biotinylated antibody in the presence of the indicated competing antibody. Heat map colors range from dark grey (100% blocking of the biotinylated antibody) to white (0% or no blocking of the biotinylated antibody)

A

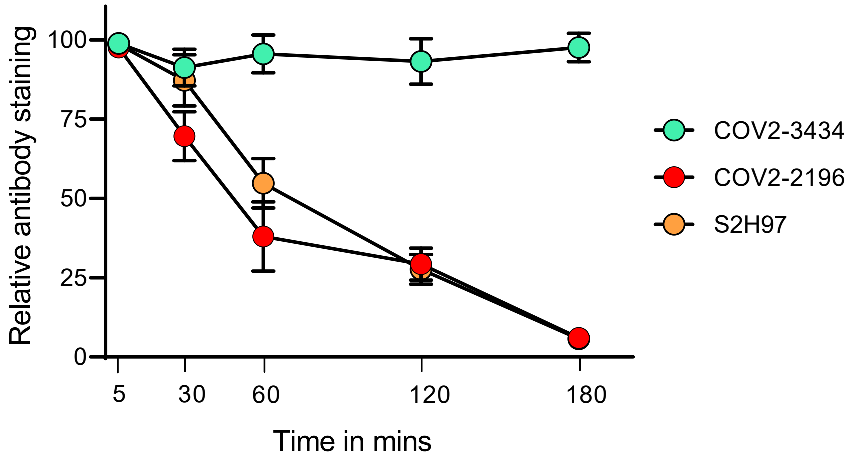

B

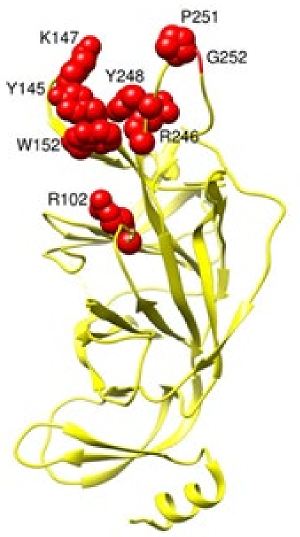

Figure S5. A. Cell-surface antibody-mediated S1 shedding. B. Epitope identification and structural characterization of COV2-3439, related to Figure 4 Residues critical for COV2-3439 binding, identified by screening COV2-3439 on an NTD alanine-scan mutagenesis library, are shown in red spheres on the NTD (PDB 7L2C).

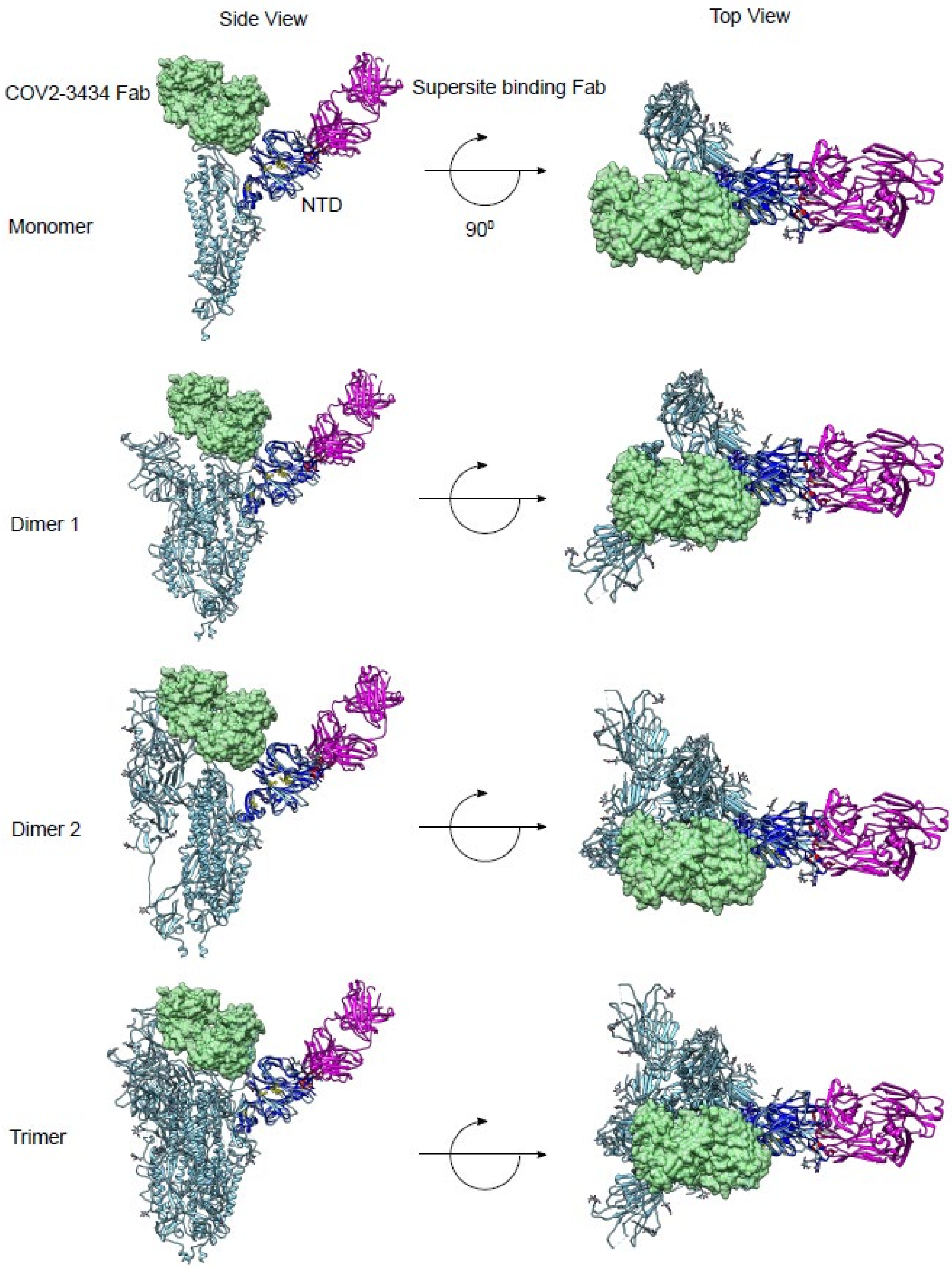

Figure S6. Structural characterization of COV2-3434, related to Figure 5 Steric clash of COV2-3434 Fab (green) with SARS-CoV2- S monomer (cyan) in open conformation when modeled double Fab (COV2-3434 Fab (green) COV2-3439 Fab (magenta) + rNTD (blue) complex on to SARS-CoV2- S monomer (cyan) in open conformation.

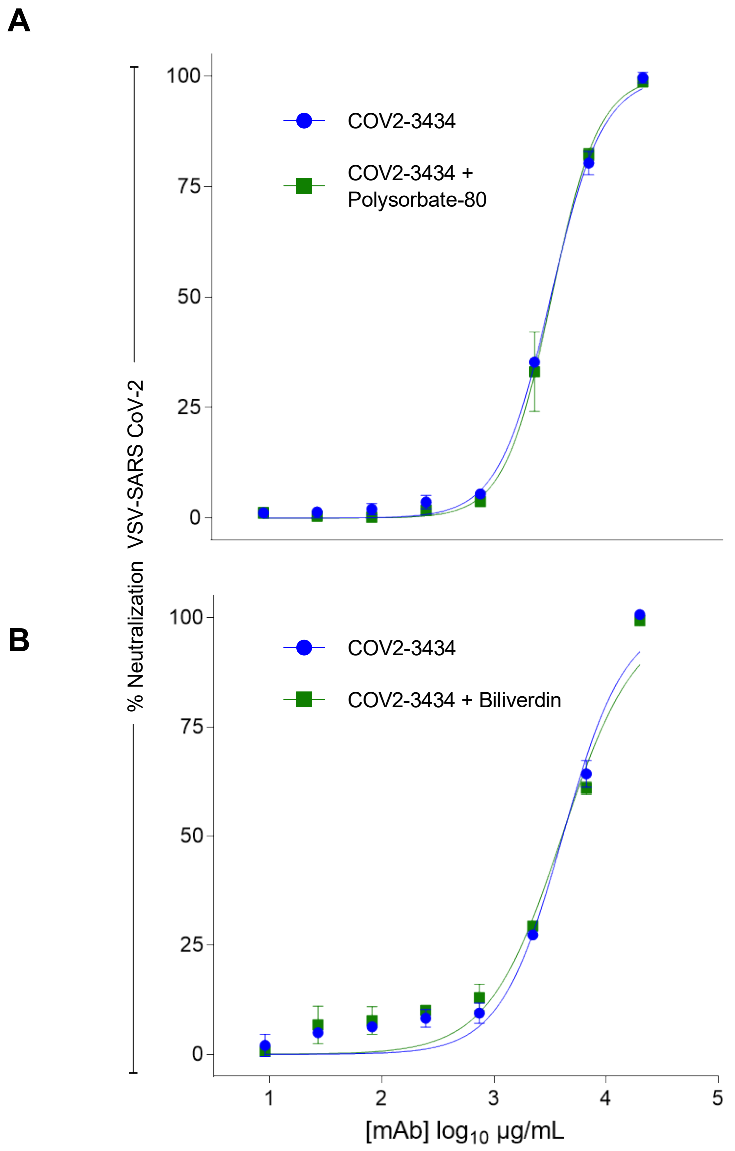

Figure S7. Neutralization of VSV-S by COV2-3434 related to Figure 5

A. Neutralization of VSV-S by COV2-3434 was measured in the absence or presence of 0.02% polysorbate-80 in Vero-CCL81 cells.

B. Neutralization of VSV-S by COV2-3434 was measured in the absence or presence of 25 µM biliverdin in Vero-CCL81 cells.

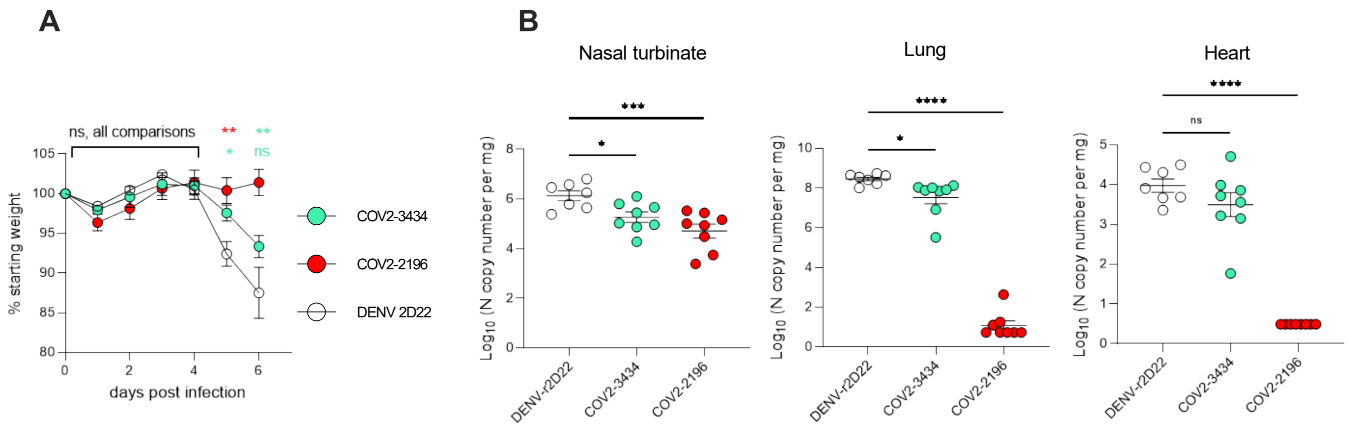

Figure S8. Protection in K18 hACE2 transgenic mice by trimer-disrupting antibody COV2-3434, related to Figure 6.

Eight-week-old female K18-hACE2 transgenic mice were inoculated by the intranasal route with 10^4 FFU of SARS-CoV-2 (WA1/2020 D614G). One day prior to virus inoculation, mice were given a single 1 mg dose of COV2-3434, COV2-2196, or isotype control mAb by intraperitoneal injection. Data are from two independent experiments, n=7 (isotype) or 8 (all other groups).
A. Weight was monitored daily. Two-way ANOVA with Dunnett's post-test with comparison to control mAb: **, $p<0.001$; *, $p<0.05$; ns, not significant.
B. At 6 dpi, tissues were collected, and viral RNA levels in indicated tissues were determined (line indicates median). One-way ANOVA with Dunnett's post-test: ****, $p<0.0001$; *$p<0.05$; ns, not significant. The dotted line represents the limit of detection (LOD) of the assay.

**Table S1. Clinical features of individuals studied**				
**Exposure**	**Donor #** [vaccine (# doses)]	**Location of exposure**	**Sample from timepoint after exposure (days)**	**Symptoms**
**Infection**	1989	Nashville, TN, USA	28	Fever, chills, fatigue, cough, shortness of breath, loss of appetite
			56	
			112	
	1988	Nashville, TN, USA	16	Fever, fatigue, cough, shortness of breath, loss of appetite
			28	
			56	
	1992	Nashville, TN, USA	13	Fever, chills, fatigue, cough, shortness of breath, headache, myalgia
			28	
			56	
	1951	Beijing, China	35	Low-grade fever, cough, runny nose
			36	
			75	
**Vaccination**	269 [Pfizer (2)]	Nashville, TN, USA	*pre-vac* -24	NA
			post-vac 250	NA
	1672 [Pfizer (2)]	Nashville, TN, USA	*pre-vac* -327	NA
			post-vac 233	NA
	1801 [Pfizer (2)]	Nashville, TN, USA	*pre-vac* -773	NA
			post-vac 10	NA
**Vaccination followed by breakthrough infection**	1079 [Pfizer (1)]	Nashville, TN, USA	*pre-vac* -111	NA
			post-vac 193<br>*post-inf* 66	Fever, chills, fatigue, cough, shortness of breath, headache, myalgia, loss of smell, loss of taste
	2062 [Pfizer (2)]	Nashville, TN, USA	*pre-vac* -11	NA
			post-vac 201<br>*post-inf* 34	Fever, cough, loss of smell, headache, sore throat

NA, indicates Not applicable.

Table S2. *IGHV1-24* genes identified in our study or from published data, with CDRH3 amino acid length and sequences.

Publication	Monoclonal antibody	*IGHV*	CDRH3 length (# amino acids)	CDRH3 amino acid sequence	*IGLV*
Chi X *et al.*, 2020	4A8	*IGHV1-24*	21	ATSTAVAGTPDLFDYYYGMDV	*IGKV2-24*
Liu L *et al.*, 2020	1-68	”	21	ATGWAVAGSSDVWYYYYGMDV	*IGLV2-18*
Liu L *et al.*, 2020	1-87	”	21	ATGIAVIGPPPSTYYYYGMDV	*IGLV2-14*
This study	COV2-3443	”	21	ATAIAVAGSPEYYYYYHGMDV	*IGLV1-44*
This study	COV2-3438	”	14	AISPAIVAAGWLDP	*IGLV2-23*
Voss *et al.*, 2021	CM25	”	14	ATGPAVRRGSWFDP	*IGLV1-51*
Liu L *et al.*, 2020	2-51	”	14	ATGWAYKSTWYFGY	*IGLV2-8*
Zhang L *et al.*, 2020	FC05	”	14	ATTTPFSSSYWFDP	*IGLV1-51*
This study	COV2-3436	”	14	ATVFAIFGVVRFDY	*IGLV1-40*
This study	COV2-3439	”	15	ATSSPIVGTTGWFDP	*IGKV1-39*
This study	COV2-3437	”	12	ATGHQLLVHWFDP	*IGKV3-20*

Table S3. Summary of electron microscopy data collection and statistics SARS-CoV-2$_{ecto}$ protein in complex with human Fabs				
		**Structure of SARS-CoV-2 S6P$_{ecto}$ protein in complex with indicated Fab**		**Structure of SARS-CoV-2 rNTD in complex with**
		**Fab COV2-3434**	**Fab COV2-3439**	**Fab COV2-3434/COV-3439**
**Microscope setting**	Microscope	TF-20	TF-20	TF-20
	Voltage (kV)	200	200	200
	Detector	US-4000 CCD	US-4000 CCD	US-4000 CCD
	Magnification	50,000x	50,000x	50,000x
	Pixel size	2.18	2.18	2.18
	Exposure (e-/Å2)	30	30	30
	Defocus range (µm)	1.5 to 1.8	1.5 to 1.8	1.5 to 1.8
**Data**	Micrographs, #	319	320	300
	Particles, #	70,634	48,000	210,000
	Particles #, after 2D	16,012	33,360	90,476
	Final particles, #	16,012	18,375	90,476
	Symmetry	C1	C1	C1
